# A newly developed circadian imbalance index (CII) and risk of cardiovascular-kidney-metabolic disease in the UK biobank

**DOI:** 10.1007/s10654-026-01373-7

**Published:** 2026-02-21

**Authors:** Jing Zhang, Dat Thien Tran, Tala El Ghoul, Susanne Strohmaier, Magdalena Żebrowska, Susan Redline, Richa Saxena, Martin K. Rutter, Eva S. Schernhammer

**Affiliations:** 1 Department of Epidemiology, Center for Public Health, Medical University of Vienna, Kinderspitalgasse 15, 1090 Vienna, Austria; 2 Division of Sleep Medicine, Harvard Medical School, Boston, MA 02115 USA; 3 Division of Sleep and Circadian Disorders, Brigham and Women’s Hospital, Boston, MA 02115 USA; 4 Center for Genomic Medicine, Massachusetts General Hospital, Boston, MA 02114 USA; 5 Department of Anesthesia, Critical Care and Pain Medicine, Massachusetts General Hospital, Harvard Medical School, Boston, MA 02114 USA; 6 Centre for Biological Timing, Division of Endocrinology, Diabetes & Gastroenterology, School of Medical Sciences, Faculty of Biology, Medicine and Health, Manchester Academic Health Science Centre, University of Manchester, Manchester, M13 9PL UK; 7 Diabetes, Endocrinology and Metabolism Centre, National Institute for Health and Care Research Manchester Biomedical Research Centre, Manchester University National Health Service Foundation Trust, Manchester, M13 9WU UK; 8 Channing Division of Network Medicine, Department of Medicine, Brigham and Women’s Hospital, Harvard Medical School, Boston, MA USA; 9 Department of Epidemiology, Harvard T.H. Chan School of Public Health, Boston, MA USA

**Keywords:** Nightshift work, Chronotype, Caffeinated coffee consumption, Vitamin D, Sleep duration, Cardiovascular-kidney-metabolic health, Cardiovascular disease, Chronic kidney disease, Type 2 diabetes

## Abstract

To examine the association between combined circadian imbalance related traits and cardiovascular-kidney-metabolic (CKM) disease risk, and their potential interaction with night shift work. This study included 191,764 UK Biobank participants without major chronic diseases who were employed at baseline (2006–2010). The Circadian Imbalance Index (CII) was developed by combining several factors indicative of a propensity for circadian misalignment. One point was assigned for each of the following components if present: evening chronotype, sleep duration ≤ 6 or ≥ 9 h/day, high neuroticism (score ≥ 7), atypical caffeinated coffee consumption (0 or ≥ 5 cups/day), and vitamin D (< 50 nmol/L), resulting in a composite scale from 0 to 5. CKM outcome (type 2 diabetes, cardiovascular diseases, chronic kidney diseases) identified by ICD codes, self-reports, or death records. Cox models estimated hazard ratios (HRs) and 95% confidence intervals (CIs) for the multivariable (MV)-adjusted association between the CII and CKM risk, including effect modification by night shift work. During a median follow-up of 13.5 years (through 2022), 16,907 incident CKM cases were identified. Among participants with European ancestry, for highest versus lowest (0–1) CII, the MV-adjusted risk of CKM was 1.95 (95%CI: 1.70–2.23; *P*_trend_<0.001). A significant positive relationship between CII and CKM risk was also observed in participants of Asian (HR = 2.03, 95%CI, 1.07–3.86; *P*_trend_=0.002), but not African ancestry (HR = 1.43, 95%CI, 0.67–3.06; *P*_trend_=0.35). Risks were higher in shift and night workers than day workers. Among Europeans, the HR for highest CII combined with current night shift work was 2.22 (95%CI, 1.95–2.53), with significant additive interaction (*P* < 0.05). In this large prospective study, Circadian Imbalance Index (CII) was associated with higher CKM risk in Europeans and Asians. Among Europeans, high CII plus night shift work posed the greatest risk.

**Supplementary Information:**

The online version contains supplementary material available at 10.1007/s10654-026-01373-7.

## Introduction

Internal biological clocks are endogenous regulators that optimally align physiology and behavior with the solar day, following an intrinsic circadian rhythm [1]. The molecular mechanisms of the clock regulate nearly every aspect of human physiology, including the sleep-wake cycle, core body temperature, secretion of hormones (e.g., cortisol and melatonin), and behavioral factors such as cognition and mood [2].

Circadian imbalance i.e. a propensity to circadian misalignment is widespread, impacting an increasing number of people. Circadian misalignment is not limited to individuals who work non-daytime schedules [3]; rather, it reflects inconsistencies between internal biological rhythms and external environmental or behavioral cues, and is influenced by factors such as chronotype [4], sleep timing [5], personality traits notably neuroticism [6], caffeine intake [7], and vitamin D levels, which are biologically linked with melatonin pathways and serve as a proxy for outdoor sunlight exposure [8, 9]. Neuroticism has been consistently correlated with shift work tolerance [10] and studies have demonstrated greater variability in sleep-wake patterns among individuals high in neuroticism [6]. Furthermore, there is accumulating observational evidence that an evening chronotype [11], short or long sleep duration [12], neuroticism [13], atypical coffee consumption [14], and vitamin D insufficiency [15] each are associated with an increased risk of cardio-metabolic diseases. Prior research has primarily assessed these circadian-related traits in isolation, without accounting for their combined effects on cardio-metabolic disease risk.

In the past few decades, the population of shift workers has increased tremendously. Today, up to 18% of workers in the European region reported working shifts [16]. Shift work, especially night shifts characterized by irregular sleep patterns and atypical exposure to environmental light, represents a pronounced form of circadian misalignment [17]. This disruption of circadian rhythms has been associated with a range of adverse health outcomes including cardio-metabolic disease [18, 19], breast and prostate cancer [20, 21], and chronic kidney disease [22]. However, research examining the interaction between shift work and circadian-related traits such as sleep duration or chronotype in relation to cardiovascular disease remains limited and yields inconsistent findings [23, 24].

In 2022, circulatory diseases accounted for close to one-third of all deaths in the European Union [25]. Cardiovascular diseases frequently coexist with type 2 diabetes and chronic kidney disease, collectively termed cardiovascular-kidney-metabolic (CKM) diseases, reflecting shared underlying mechanisms including dysglycemia, dyslipidemia, hypertension, and obesity [26, 27]. When one system is impaired, it can exacerbate dysfunction in the other, increasing the risk of subsequent health complications and mortality [28]. To our knowledge, no prior study has assessed the link between a composite score for circadian misalignment and risk for incident CKM, or how night shift work influences their relationship [29].

Using data from the UK Biobank, we evaluated the association between a combination of circadian imbalance-related factors, characterized by a novel Circadian Imbalance Index (CII), and the risk of CKM disease, in the whole cohort and when stratified by the night shift work status.

## Methods

### Study cohort

The UK Biobank is a large-scale prospective study, which began in 2006, enrolling half a million residents of the United Kingdom, aged 40 to 69 years [30]. All participants completed an initial baseline assessment, which included sociodemographic psychosocial, physical, and lifestyle information, as well as the collection of biological samples [30]. The UK Biobank can track participants’ health outcomes through various national datasets, including Hospital Episode Statistics and national death and cancer registries. The National Research Ethics Service approved the UK Biobank study (ref. 11/NW/0382), and all participants provided written informed consent.

### Assessment of circadian imbalance related factors

The UK Biobank collected data on chronotype, sleep duration, neuroticism, and caffeinated coffee consumption through a baseline self-response questionnaire. Specifically, chronotype preference was assessed using the question, ‘Do you consider yourself to be?’ with responses of (1) definitely a ‘morning’ person, (2) more a ‘morning’ than ‘evening’ person, (3) more an ‘evening’ person than a ‘morning’ person, (4) definitely an ‘evening’ person, (5) do not know. Sleep duration was self-reported through the question ‘About how many hours sleep do you get in every 24 hours? (Please include naps)’. Neuroticism was assessed using 12 questions from the Eysenck Personality Inventory Neuroticism Scale (EPIN-R) [31]. The neuroticism score was calculated by summing the number of “Yes” responses across these questions, resulting in a single integer score for each participant ranging from 0 to 12. Consumption of caffeinated coffee was assessed using two dietary questions. Participants were first asked, ‘How many cups of coffee do you drink each DAY? (Include decaffeinated coffee)’. Response options included a specific number of cups, ‘<1’, ‘do not know’ or ‘Prefer not to answer’. For those who reported drinking at least one cup of coffee per day, a follow-up question was asked: ‘What type of coffee do you usually drink?’ with response options of ‘Decaffeinated coffee (any type)’, ‘Instant coffee’, ‘Ground coffee (include espresso, filter etc.)’ or ‘other type of coffee’. For our analysis, we categorized caffeinated coffee consumption into the following groups: 0, 1, 2, 3, 4, >=5 cups/day, with participants reporting decaffeinated coffee intake classified into the 0 cups/day group. Serum concentrations of 25(OH)D (nmol/L) were determined using the chemiluminescent immunoassay method (DiaSorin Liaison XL platform by DiaSorin S.p.A). Detailed information regarding the measurement of biochemical markers and quality assessment are provided elsewhere [32, 33].

### Construction of circadian imbalance index (CII)

We conducted literature searches and relied on previously published systematic reviews [8, 29, 34] and reports [35] to identify factors of potential interest for our definition of circadian imbalance. Based on relevance to the circadian system and availability within the UK Biobank, we chose a set of five circadian imbalance-related factors (chronotype, sleep duration, neuroticism, caffeinated coffee intake, and serum vitamin D concentration) to derive a Circadian Imbalance Index (CII). Specifically, morning chronotypes have previously been associated with better circadian alignment whereas evening chronotype are more prone to misalignment [36]. Additionally, both short (< 7 h) and long (> 9 h) sleep durations have been linked to increased circadian imbalance [37]. Individuals with high neuroticism tend to report greater variability in sleep-wake patterns and appear more susceptible to misalignment [6]. Moderate caffeine consumption may support more stable circadian alignment by enhancing the internal clock’s responsiveness to environmental cues [7]. Low vitamin D levels have also been associated with circadian misalignment, potentially due to its role in melatonin biosynthesis and signaling pathways that regulate the sleep-wake cycle [8, 38].

For each factor, participants were assigned one point if they exhibited the following characteristics: evening chronotype (including “definitely an ‘evening’ person” or “more an ‘evening’ person than a ‘morning’ person”) [39]; short or long sleep duration (≥ 9 h or ≤ 6 h/day) [40]; high neuroticism score (≥ 7) [31]; low serum vitamin D concentration (< 50 nmol/L) [41]; and atypical caffeinated coffee intake (none or ≥ 5 cups/day, approximately 400 mg caffeine) [42]. Notably, given that approximately 80% of the global population consumes caffeine daily [43], complete avoidance of caffeinated coffee may reflect underlying caffeine sensitivity rather than a typical behavioral pattern. Participants with alternative expressions of these factors were assigned a zero for each respective category. The CII was calculated by summing the points across all five factors and ranged from 0 to 5, with a higher index indicating greater propensity to circadian imbalance. For interaction analyses, we categorized individuals into three circadian imbalance groups: ‘low CII’ (0 ≤ CII ≤ 1); ‘intermediate CII’ (2 ≤ CII ≤ 3); and ‘high CII’ (4 ≤ CII ≤ 5).

### Assessment of night shift work

At baseline from 2006 to 2010, participants who were in paid employment or self-employed were asked whether their primary job involved shift work, defined as work schedules outside typical daytime hours (9am-5pm). Participants who answered ‘yes’ were further asked if their job included night shifts, characterized by working during normal sleeping hours (12am to 6am). Responses to the two questions included the following options: ‘never/rarely’, ‘sometimes’, ‘usually’, ‘always’, along with ‘prefer not to answer’ and ‘do not know’. Based on these responses and consistent with existing research [44], participants were categorized into one of the following work status groups: ‘day workers’, ‘shift workers’ (i.e., those who never or rarely worked night shifts), and ‘night shift workers’ (i.e., those who reported working sometimes, usually, or always night shifts).

To further assess their lifetime employment history, between July and September 2015, all participants were invited via email to complete an online occupational history questionnaire. A subset of participants responded, providing detailed employment histories on all jobs they had held, including the duration of each job and details about shift work schedules. Using this data and findings from previous studies, we calculated three key night shift work metrics: duration of night shift work (total number of years spent working night shifts), cumulative exposure (total number of night shifts over a lifetime), and intensity of night shift work (average number of night shifts per month) [45, 46].

### Assessment of outcome

The primary outcome was defined as time to any incident CKM disease, which included type 2 diabetes (T2D), cardiovascular disease (CVD) or chronic kidney disease (CKD). These outcomes were identified through the national death registry, hospital inpatient records and self-reports. The national death registry and hospital impatient records were classified based on the 10th edition of the International Classification of Diseases (ICD-10) [47] as follows: type 2 diabetes: E11 (non-insulin-dependent diabetes mellitus); cardiovascular disease: I50 (heart failure), I21-I25 (ischemic heart disease) and I60-I64 (stroke); chronic kidney disease: N18 (kidney failure) and I12-I13 (hypertensive renal disease) [48].

### Assessment of covariables

We considered a range of demographic, health and lifestyle factors [45, 49] that were all assessed at baseline, as potential confounders in our analyses. They included age, sex, average total household income before tax, education level, recruitment season, smoking status, alcohol consumption, physical activity, body mass index (BMI), hypertension and elevated cholesterol. For missing covariable data, we applied sex-specific median values for continuous variables and introduced a missing indicator for categorical variables. To ensure accurate ancestry classification, we based our classification on detailed genetically derived, rather than self-reported ancestry. Ancestry estimates are described in the Genetic Ancestry Assessment Supplement. A detailed description of all other covariables is provided in Supplemental Table 1.

### Analytic sample

From the total UK Biobank cohort, we excluded individuals with prevalent cancer or CKM disease at baseline (*N* = 69,562), as well as those with missing responses – including “prefer not to answer” – for circadian imbalance related variables (*N* = 124,505). Additionally, participants who were not in paid employment or self-employed at baseline (*N* = 116,447) were excluded. These exclusions resulted in a final analytic sample of 191,764 individuals for the analysis considering current night shift exposure. Among these, a subset of 47,843 participants of European ancestry completed an online employment history questionnaire in 2015, which was used to assess lifetime exposure to night shift work (Supplemental Fig. 1). In analyses using this subset, participants of non-European ancestry were excluded due to limited case numbers.

### Statistical analyses

The follow-up period extended from the date of enrollment (2006–2010) until the first occurrence of either a diagnosis of CKM disease or a censoring event, which included death, withdrawal from the study, or the end of the designated follow-up period. Region-specific designated follow-up end dates were applied: October 31, 2022, for England, August 31, 2022, for Wales and May 31, 2022, for Scotland. To assess the relationship between the CII and incidence of CKM, we employed Cox proportional hazards models to estimate hazard ratios (HRs) and 95% confidence intervals (CIs). The proportional hazards assumption was tested for all models using Schoenfeld residuals and no indications of violations were observed (Supplemental Fig. 2 for main model).

The main analyses were conducted stratified by three main genetic ancestry groups (European, Asian and African). In addition to stratifying, in sensitivity analyses, we also adjusted for ethnicity in the overall analytic sample; however, due to the predominance of European ancestry (87%), results were highly similar to those in the stratum of participants with European ancestry only. Guided by prior research [45, 49], we considered four models serially adjusting for potential covariables: Model 1 accounted for age and sex; model 2 additionally included socioeconomic indicators, specifically average total household income before tax and education level; model 3 further incorporated lifestyle factors, including smoking status, alcohol consumption, and physical activity. Finally, to account for its potential mediating role in the CII-CKM relationship [50], BMI was introduced separately in model 4. Although these potential confounders and mediators were assessed at baseline only, it appears reasonable to assume that they would likely remain on the same trajectory through follow-up. Further adjustment for psychosocial factors and depressive symptoms did not alter our estimated and we therefore did not retain these variables in our main models. To evaluate potential linear trends, the CII was additionally analyzed as a continuous variable in various models as specified above. We also performed cause-specific analyses considering three individual diseases of CKM disease as competing events.

In subgroup analyses, we first performed sex-stratified analyses within the overall study population. Among the women, we further considered menopausal status as a potential confounder but because results remained virtually unchanged, did not retain it in our models. Subsequently, among individuals of European ancestry, we performed additional analyses stratified by current work status (as reported in the baseline questionnaire) and lifetime exposure to night shift work (as assessed in the 2015 occupational questionnaire). To explore whether the associations between CII and CKM disease risk varied according to night shift work status, participants were categorized into nine groups based on the combination of night shift work status (day worker, shift worker and night shift worker) and CII level (low, intermediate and high). HRs for incident CKM disease were then estimated for each group, using dayworkers with low CII as the reference category. To assess potential multiplicative interaction, we included a product term of CII group and night shift work status or available lifetime-employment history data in the regression models and compared the − 2 log-likelihood values of models with and without the inclusion of a product term. To evaluate additive interaction [51], we calculated the relative excess risk to interaction (RERI) and the attributable proportion (AP), along with their corresponding 95% confidence intervals. Details regarding the formulas used for assessing additive interaction are provided in the Supplementary Statistical Methods. Finally, we constructed the CII weighted to the coefficients derived directly from the association between each CII component and the CKM disease outcome; results remained largely unchanged and we retained the unweighted score as it promotes the applicability and generalizability of the CII across various health outcomes rather than being restricted to the weights specific to CKM disease. All statistical analyses were performed using R software, version 4.3.1 (R foundation for Statistical Computing). All P-values were two-sided, with a threshold of *P* < 0.05 considered statistically significant.

## Results

### Population characteristics

Table 1 presents baseline characteristics of the study participants by level of CII. Among the 191,764 participants, 66,962 (34.9%), 65,765 (34.3%), 42,246 (22.0%), 14,468 (7.5%), and 2,323 (1.2%) had a CII of 0–1, 2, 3, 4, and 5, respectively. The mean age of participants was 52 years (SD = 7), 51% were women, 87% were of European ancestry, and 83% were employed in non-shift work occupations. Overall, participants with higher CII were more likely to be women, to undertake night shift work, and have lower educational attainment and household income. They were also more likely to currently smoke, to exercise less, and to have a higher BMI.

**Table 1:** Characteristics of 191,764 participants from the UK biobank*, overall and according to category of circadian imbalance index (CII)

Baseline Characteristic	Overall	Circadian Imbalance Index				
		0–1	2	3	4	5
**Number of participants**	191,764	66,962	65,765	42,246	14,468	2,323
**Female % (N)**	50.84 (97,490)	48.19 (32,271)	50.95 (33,506)	52.90 (22,350)	55.45 (8,023)	57.68 (1,340)
**Age (years) (mean(SD))**	52.36 (7.03)	52.97 (7.10)	52.37 (7.01)	51.84 (6.95)	51.29 (6.79)	50.59 (6.71)
**Ethnicity % (N)**						
African	1.35 (2,583)	0.45 (333)	1.36 (897)	2.24 (946)	2.41 (349)	2.50 (58)
Asian	1.82 (3,481)	0.84 (562)	2.05 (1,350)	2.61 (1,103)	2.75 (398)	2.93 (68)
European	86.67 (166,194)	89.49 (59,921)	86.21 (56,698)	84.27 (35,600)	83.34 (12,058)	82.52 (1,917)
Other	10.17 (19,506)	9.178 (6,146)	10.37 (6,820)	10.88 (4,597)	11.49 (1,663)	12.05 (280)
**Night shift work status % (N)**						
Day workers	83.47 (160,068)	86.06 (57,629)	83.78 (55,096)	81.30 (34,347)	78.17 (11,309)	72.62 (1,687)
Shift workers	8.10 (15,529)	7.25 (4,856)	8.09 (5,323)	8.78 (3,709)	9.52 (1,378)	11.32 (263)
Night shift workers	8.43 (16,167)	6.69 (4,477)	8.13 (5,346)	9.92 (4,190)	12.31 (1,781)	16.06 (373)
**Current smokers % (N)**	10.37 (19,892)	7.707 (5,161)	10.18 (6,697)	12.51 (5,283)	15.97 (2,311)	18.94 (440)
**Daily or almost daily drinker % (N)**	20.07 (38,489)	21.94 (14,691)	19.64 (12,915)	18.62 (7,868)	18.14 (2,625)	16.79 (390)
**Household income % (N)**						
< £18,000	8.35 (16,016)	6.52 (4,365)	8.18 (5,378)	9.89 (4,180)	12.17 (1,761)	14.29 (332)
£18,000 ~ £100,000	76.07 (145,874)	76.45 (51,191)	76.05 (50,017)	76.06 (32,132)	74.58 (10,790)	75.08 (1,744)
> £100,000	7.64 (14,644)	9.20 (6,158)	7.75 (5,095)	6.09 (2,573)	5.11 (739)	3.40 (79)
Unknown	7.94 (15,230)	7.84 (5,248)	8.02 (5,275)	7.96 (3,361)	8.14 (1,178)	7.23 (168)
**College education % (N)**	39.19 (75,151)	41.05 (27,487)	39.78 (26,163)	37.18 (15,707)	34.82 (5,038)	32.54 (756)
**Physical activity % (N)**						
Low (< 10 MET-h/week)	15.94 (30,570)	13.09 (8,767)	16.31 (10,728)	18.28 (7,722)	19.64 (2,842)	22.00 (511)
Middle (10 ~ 50 MET-h/week)	43.08 (82,603)	44.86 (30,038)	43.31 (28,486)	41.75 (17,639)	38.75 (5,606)	35.90 (834)
High (> 50 MET-h/week)	24.77 (47,504)	27.49 (18,409)	24.22 (15,925)	22.51 (9,509)	21.89 (3,167)	21.27 (494)
Unknown	16.21 (31,087)	14.56 (9,748)	16.16 (10,626)	17.46 (7,376)	19.72 (2,853)	20.84 (484)
**Hypertension % (N)**	41.16 (78,939)	41.58 (27,842)	41.32 (27,177)	40.68 (17,186)	40.30 (5,831)	38.87 (903)
**Self-report hyperlipidemia % (N)**	7.13 (13,668)	6.83 (4,570)	7.04 (4,628)	7.49 (3,164)	7.76 (1,123)	7.88 (183)
**BMI (kg/m** ^**2**^ **) (mean(SD))**	27.15 (4.63)	26.59 (4.15)	27.21 (4.64)	27.58 (4.92)	27.97 (5.21)	28.69 (5.78)

BMI: body mass index; SD: standard deviation. *No restrictions based on ethnicity were implemented

### Prospective association between CII and CKM disease risk

During a median follow-up period of 13.5 years, a total of 16,907 incident cases of CKM disease were documented. Table 2 presents the associations between CII and CKM disease risk across genetic ancestry groups, as estimated by multivariable-adjusted models. Among individuals of either European or Asian ancestry, higher CII values were consistently associated with a higher risk of CKM disease compared to those with a CII of 0–1, across all models (*P*_*trend*_ < 0.05). Following adjustments for age, sex, household income and education level in Model 2, the HRs for Europeans and Asians were slightly attenuated but remained statistically significant. Specifically for the Europeans, the HRs (95% CI) for CII values of 2, 3, 4, and 5 were 1.26 (1.21–1.31), 1.42 (1.36–1.49), 1.65 (1.55–1.76), and 1.95 (1.70–2.23), respectively. Among Asian participants, compared with those with a CII of 0–1, the multivariable-adjusted HRs (95% CI) for CII of 2 to 5 were 1.41 (1.07–1.86), 1.49 (1.13–1.97), 1.64 (1.17–2.31), and 2.03 (1.07–3.86), respectively, in Model 2. Further adjustment for smoking status, alcohol consumption, and physical activity (Model 3), as well as body mass index (Model 4), attenuated these associations somewhat, yet they remained significant among Europeans and Asians. In contrast, no significant association was observed between CII and CKM disease risk among participants of African ancestry (Supplemental Table 17).

**Table 2:** Prospective associations between circadian imbalance index (CII) and risk of incident cardiovascular-kidney-metabolic disease among participants from the UK Biobank, stratified by European and Asian ancestries

Ethnicity	CII	Case/*N*	Absolute event rates	Model 1^a^		Model 2^b^		Model 3^c^		Model 4^d^	
			(Events/1000PY)	HR (95%CI)^1^	*P*_trend_	HR (95%CI)	*P*_trend_	HR (95%CI)	*P*_trend_	HR (95%CI)	*P*_trend_
European (*N* = 166,194)	0–1	4 474/59 921	5.92	Reference	< 0.001	Reference	< 0.001	Reference	< 0.001	Reference	< 0.001
	2	4 979/56 698	7.00	1.27(1.22–1.32)		1.26(1.21–1.31)		1.22(1.17–1.27)		1.14(1.09–1.18)	
	3	3 394/35 600	7.61	1.47(1.40–1.53)		1.42(1.36–1.49)		1.34(1.28–1.40)		1.21(1.16–1.27)	
	4	1 269/12 058	8.43	1.74(1.63–1.85)		1.65(1.55–1.76)		1.53(1.44–1.63)		1.34(1.26–1.42)	
	5	221/1 917	9.29	2.08(1.82–2.38)		1.95(1.70–2.23)		1.75(1.53–2.01)		1.42(1.24–1.63)	
Asian (*N* = 3,481)	0–1	67/562	9.64	Reference	< 0.001	Reference	0.002	Reference	0.006	Reference	0.024
	2	211/1 350	12.85	1.42(1.08–1.87)		1.41(1.07–1.86)		1.39(1.06–1.84)		1.37(1.04–1.80)	
	3	183/1 103	13.80	1.51(1.14–2.00.14.00)		1.49(1.13–1.97)		1.44(1.09–1.91)		1.36(1.03–1.81)	
	4	68/398	14.29	1.71(1.22–2.39)		1.64(1.17–2.31)		1.58(1.13–2.23)		1.49(1.06–2.09)	
	5	11/68	13.48	2.13(1.12–4.05)		2.03(1.07–3.86)		1.91(1.00–3.64.00.64)		1.82(0.95–3.48)	

^a^Model 1 includes sex and age

^b^Model 2 includes variables in Model 1 and household income and education

^c^Model 3 includes variables in Model 2 and smoking status, alcohol consumption and physical activity

^d^Model 4 includes variables in Model 3 and body mass index (BMI)

^1^HR = Hazard Ratio, CI = Confidence Interval

In sensitivity analyses, we additionally adjusted for hypertension and hyperlipidemia, as well as recruitment season (Supplemental Table 2); excluded those with missing information on any of the covariables that were considered (Supplemental Table 3); and excluded participants with a report of incident CKM within the first 2 years and 5 years of follow-up (Supplemental Table 4a and Supplemental Table 4b); all results consistently demonstrated that higher CII levels were associated with higher risks of CKM diseases. Further, in analysis adjusting (rather than stratifying) for genetic ethnicity results were similar to those in the stratum of participants with European ancestry only (Supplemental Table 5). In addition, we found that each individual circadian trait was independently associated with an elevated risk of CKM disease. Moreover, within the European ancestry group, the overall CII was significantly associated with the risk of each individual CKM disease component. Detailed results of these analyses are provided in Supplemental Tables 12–15, and Supplemental Fig. 3. Results from the cause-specific analyses remained largely consistent with main result (Supplementary Table 16).

Although in gender-stratified analyses, the association between CII and CKM disease risk appeared stronger among women compared to men in Model 2, there was no statistically significant effect modification by gender (*P*_interaction_= 0.666) (Supplemental Table 6). Further stratification by genetic ancestry (Supplemental Table 7) revealed that the HRs in Model 2 were particularly elevated among European women and Asian men. No significant associations were observed within African ancestry gender subgroups.

### Additive and multiplicative interactions of CII and night shift work

Among individuals of European ancestry, a significant dose-response relationship between the CII and CKM disease risk was observed across all categories of work status, with both shift workers and night shift workers exhibiting elevated CKM disease risk (Table 3). Specifically, compared to participants with a CII of 0–1, the multivariable-adjusted HRs (95%CIs) for those with CII of 5 in Model 2 were 1.82 (1.54–2.15) among day workers, 2.57 (1.82–3.62) among shift workers, and 1.86 (1.34–2.57) among night shift workers. Consistent patterns of significant associations between CII and CKM were also observed in a subset of participants who provided detailed lifetime occupational histories, particularly among those who had worked night shifts for over 20 years or engaged in night shift work for at least 8 nights per month (Supplemental Tables 8–9).

**Table 3:** Prospective associations between circadian imbalance index (CII) and risk of incident cardiovascular-kidney-metabolic disease among 166,194 European ancestry participants from the UK biobank, stratified by work status

Group	CII	Case/*N*	Model 1^a^		Model 2^b^		Model 3^c^		Model 4^d^	
			HR (95%CI)^1^	*P*_trend_	HR (95%CI)	*P*_trend_	HR (95%CI)	*P*_trend_	HR (95%CI)	*P*_trend_
Day workers (*N* = 139,853)	0–1	3 761/51 699	Reference	< 0.001	Reference	< 0.001	Reference	< 0.001	Reference	< 0.001
	2	4 020/47 953	1.25 (1.19–1.30)		1.23 (1.18–1.29)		1.20 (1.15–1.25)		1.12 (1.07–1.17)	
	3	2 634/29 267	1.43 (1.36–1.50)		1.39 (1.32–1.46)		1.32 (1.25–1.38)		1.19 (1.13–1.25)	
	4	930/9 525	1.66 (1.55–1.79)		1.60 (1.49–1.72)		1.49 (1.38–1.60)		1.30 (1.21–1.40)	
	5	144/1 409	1.92 (1.62–2.26)		1.82 (1.54–2.15)		1.64 (1.39–1.94)		1.36 (1.15–1.61)	
Shift workers (*N* = 13,029)	0–1	377/4 236	Reference	< 0.001	Reference	< 0.001	Reference	< 0.001	Reference	< 0.001
	2	453/4 407	1.25 (1.09–1.43)		1.24 (1.08–1.42)		1.19 (1.04–1.37)		1.12 (0.98–1.29)	
	3	373/3 036	1.58 (1.37–1.83)		1.54 (1.34–1.78)		1.46 (1.26–1.69)		1.33 (1.15–1.53)	
	4	132/1 137	1.59 (1.31–1.95)		1.55 (1.27–1.89)		1.41 (1.15–1.72)		1.25 (1.02–1.53)	
	5	36/213	2.68 (1.90–3.78)		2.57 (1.82–3.62)		2.34 (1.66–3.31)		1.88 (1.33–2.66)	
Night shift workers (*N* = 13,312)	0–1	336/3 986	Reference	< 0.001	Reference	< 0.001	Reference	< 0.001	Reference	< 0.001
	2	506/4 338	1.46 (1.27–1.68)		1.45 (1.26–1.66)		1.40 (1.22–1.61)		1.28 (1.11–1.47)	
	3	387/3 297	1.51 (1.31–1.75)		1.48 (1.28–1.71)		1.39 (1.20–1.61)		1.22 (1.05–1.41)	
	4	207/1 396	2.01 (1.69–2.39)		1.93 (1.62–2.30)		1.81 (1.52–2.15)		1.60 (1.34–1.90)	
	5	41/295	1.94 (1.40–2.68)		1.86 (1.34–2.57)		1.65 (1.19–2.29)		1.29 (0.93–1.79)	

^a^Model 1 includes sex and age

^b^Model 2 includes variables in Model 1 and household income and education

^c^Model 3 includes variables in Model 2 and smoking status, alcohol consumption and physical activity

^d^Model 4 includes variables in Model 3 and body mass index (BMI)

^1^HR = Hazard Ratio, CI = Confidence Interval

*‘Shift workers’ were those who worked shift work, but never or rarely worked night shifts, ‘Night shift workers’ were those who report working night shifts sometimes, usually, or always

**Fig. 1:**
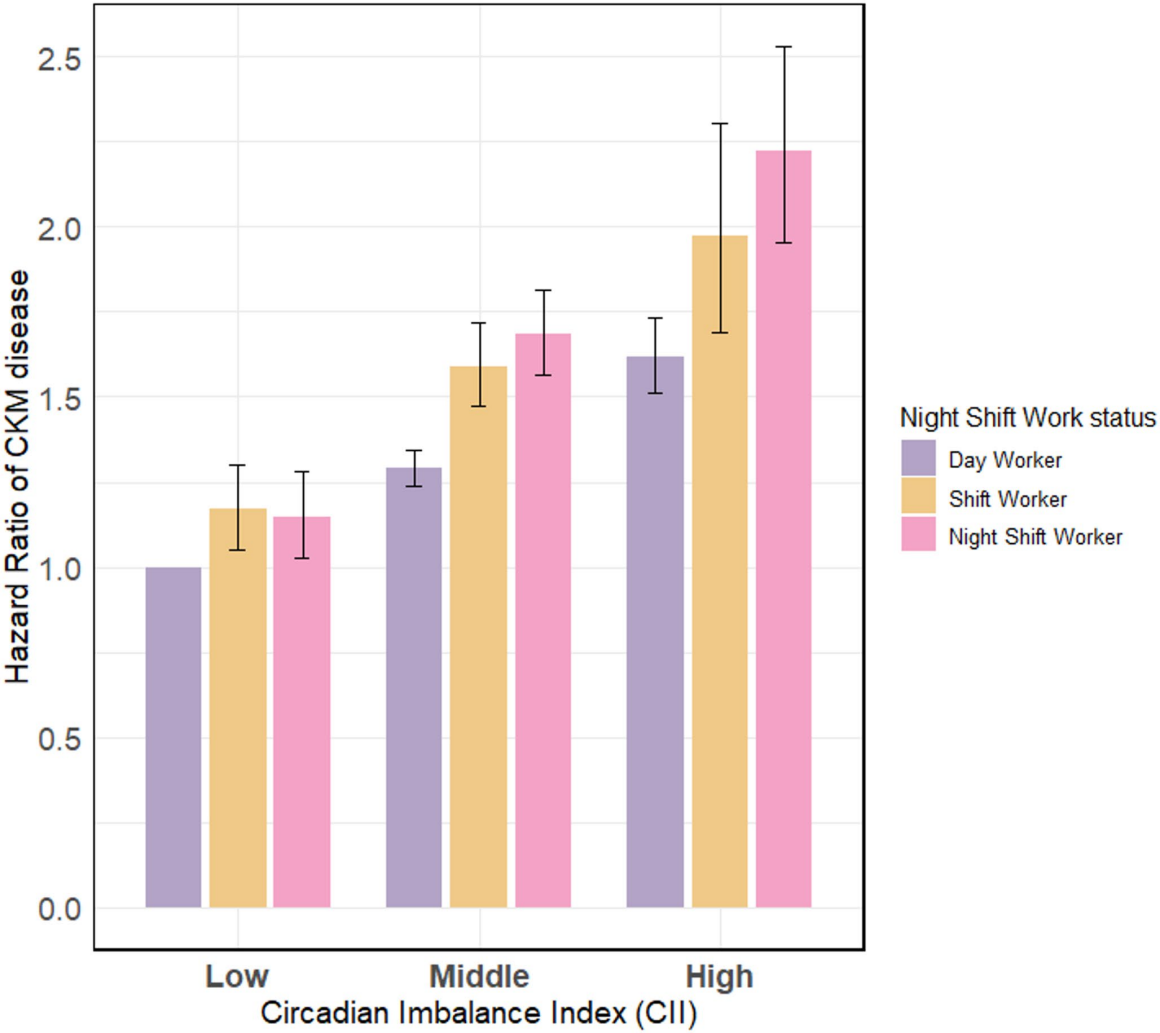
Multivariable adjusted HRs and 95% CIs of cardiovascular-kidney-metabolic disease according to joint categories of the Circadian Imbalance Index (CII) and night shift work status for European ancestry participants, in multivariable model adjusted for sex, age, household income and education, *n* = 166,194. (Cases number of dayworker with low CII: 3,761; shift worker with low CII: 377; night shift worker with low CII: 336; dayworker with middle CII: 6,654; shift worker with middle CII: 826; night shift worker with middle CII: 893; day worker with high CII: 1,074; shift worker with high CII: 168; night shift worker with high CII: 248)

We further assessed the joint association of night shift work status and CII categories with the CKM disease outcome. Within each work status group, a higher CII was consistently associated with an elevated risk of CKM disease in a dose-response manner. Specifically, compared to the reference group (day workers with low CII of 0–1), night shift workers with an intermediate CII (HR: 1.69; 95% CI: 1.57–1.81) or a high CII (HR: 2.22; 95% CI: 1.95–2.53) exhibited significantly elevated risk of CKM disease. A similarly elevated HR was also observed among shift workers with intermediate or high CII (Fig. 1; Table 4). Although the test for multiplicative interaction was not statistically significant (*P*_interaction_ = 0.238), we observed a significant additive interaction between night shift work and both intermediate (2–3) or high CII (4–5) (Table 4). Specifically, for night shift workers with a high CII, the relative excess risk due to interaction (RERI) was estimated at 0.456 (95% CI: 0.138–0.775), and the attributable proportion (AP) was 0.205 (95% CI: 0.083–0.327) (Table 4). These findings indicate a 45.6% excess relative risk attributable to the additive interaction between night shift work and high CII, with 20.5% (95% CI: 8.3–32.7%) of the CKM disease risk in this group being attributable to their combined effect (Table 4). Furthermore, similar patterns of joint associations were observed when other metrics of night shift work, including duration and intensity, were considered (Supplemental Fig. 4, Supplemental Tables 10–11). Notably, significant additive interactions were also observed between a high CII and long-term night shift work (≥20 years), as well as between a high CII and high-intensity night shift work (≥ 8 nights per month) (Supplemental Tables 10, 11).

**Table 4:** Multivariable adjusted HR with 95% CI, RERI and AP for additive interaction between circadian imbalance index (CII) and shift work status for cardiovascular-kidney-metabolic disease among European ancestry UKB participants, stratified by categories of CII and night shift work status, *n* = 166,194

Characteristic	*N*	Case	HR (95% CI)^1^	*P* value	RERI (95%CI)^2^	AP (95%CI)^3^
Low CII (0–1)						
Day workers	51,699	3,761	Reference		Ref.	Ref.
Shift workers	4,236	377	1.17 (1.05, 1.30)	0.004	-	-
Night Shift Workers	3,986	336	1.15 (1.03, 1.28)	0.016	-	-
**Middle CII (2–3)**						
Day workers	77,220	6,654	1.29 (1.24, 1.34)	< 0.001	-	-
Shift workers	7,443	826	1.59 (1.48, 1.72)	< 0.001	0.132 (−0.034–0.298)	0.083 (−0.018–0.184)
Night Shift Workers	7,635	893	1.69 (1.57, 1.81)	< 0.001	**0.249 (0.079–0.419)**	**0.148 (0.052–0.243)**
**High CII (4–5)**						
Day workers	10,934	1,074	1.62 (1.51, 1.73)	< 0.001	-	-
Shift workers	1,350	168	1.98 (1.69, 2.31)	< 0.001	0.185 (−0.152–0.522)	0.094 (−0.065–0.252)
Night Shift Workers	1,691	248	2.22 (1.95, 2.53)	< 0.001	**0.456 (0.138–0.775)**	**0.205 (0.083–0.327)**

^1^HR = Hazard Ratio, CI = Confidence Interval

^2^RERI = relative excess risk due to the interaction

^3^AP = attributable proportion due to the interaction

^*^Model adjusted for sex, age, household income and education

^*^To estimate the RERI and AP, the low CII (0–1) and the day worker group were the reference categories. ‘Shift workers’ were those who work shift works, but never or rarely worked night shifts, ‘Night shift workers’ were those who report working night shifts sometimes, usually, or always

## Discussion

In this study, we observed that higher propensity for circadian imbalance, as described by our newly developed Circadian Imbalance Index (CII), was significantly associated with a higher risk of CKM disease. Moreover, compared to participants with low circadian imbalance, those with higher CII was associated with a progressively elevated risk of CKM disease across all metrics of night shift work status (duration and intensity). Participants who worked night shifts and were classified into the high CII group (4–5) exhibited the greatest risk when compared to day workers in the low CII group (0–1). Furthermore, we identified a significant additive interaction between CII categories and night shift work status on CKM risk. This interaction was particularly pronounced among individuals with extended night shift duration (≥ 20 years) and high night shift intensity (≥ 8 nights per month).

### Comparison with other studies

To the best of our knowledge, this is the first prospective cohort study to investigate the relationships between a newly developed CII, integrating evening chronotype, short or long sleep duration, scoring higher on the neuroticism spectrum, atypical caffeinated coffee intake and low serum concentration vitamin D, and the risk of CKM disease. Though prior studies have explored the associations of night shift work, chronotype, and sleep duration with various chronic health outcomes independently [52–55], the potential additive effect of multiple circadian-related traits, as captured by the CII, on the risk of CKM diseases has not been previously evaluated. One previous study found that evening chronotype was significantly associated with cardiovascular health in night-shift workers [56]. Additionally, Young et al. proposed that night shift workers with long or short sleep duration had higher blood pressure [57]. In this study, we newly constructed a CII by taking into account the combined impact of five circadian imbalance traits on CKM risk, which reflects the most comprehensive circadian imbalance evaluation to date.

We observed significant results among participants of European and Asian ancestry, but not among those of African ancestry. From the index distribution, we observed differences between Asian and European ancestries in sleep duration, caffeinated coffee consumption, and vitamin D levels, which likely reflect both genetic and social influences. Given these ancestry-specific patterns in lifestyle and biology, we aim to validate the CII in larger populations to further explore these ethnic differences. We found CII to be associated with a higher risk of CKM among the women but not the men of European ancestry in our study, whereas the opposite pattern was observed among Asian ancestry, though none of these interactions reached statistical significance, and so these results should be interpreted with caution. While a previous small experiment has suggested that circadian misalignment might have a stronger effect on metabolic disorder among women compared with men [58], gender differences regarding metabolic disorder syndrome have been mixed in numerous countries [59]. Alternatively, there may be gender differences in the types of occupations held by men and women across different regions of the world, particularly with respect to the intensity and nature of shift work, including night shifts. Overall, these secondary gender-ancestry subgroup analyses require further exploration.

We observed a higher risk of CKM disease with increasing CII among night shift workers, especially among those with longer lifetime duration in terms of years worked night shifts, and greater intensity of night shifts. Similar to our finding, several previous studies assessing more detailed metrics of night shift work have described an increased risk of type 2 diabetes [45] or cardiovascular disease [53] with longer duration or greater intensity of night shift work.

Our findings highlight the additive interaction between night shift work status and CII on CKM disease risk. Specifically, the combination of both night shift work and high CII would result in an additional 20.5% of CKM cases. Consistently, we also observed that the significantly higher risk of CKM disease associated with longer lifetime duration or greater intensity of night shift work was further amplified among individuals in the high CII group. These findings raise the possibility that keeping a low CII among night shift workers, especially those with long-term or high-intensity night shift exposure, may be an effective prevention strategy for CKM disease risk. From a public health perspective, the CII offers a valuable research framework for evaluating the degree of circadian imbalance in individuals, in particular among night shift worker. By systematically assessing circadian imbalance, the CII may help identify those at higher risk for CKM, potentially supporting the investigation of targeted interventions. Beyond occupational health, this approach has broader relevance for the general population, as modern lifestyles—such as irregular sleep patterns, late-night screen exposure, and social jet lag—can also lead to circadian misalignment. Ultimately, the CII can inform the development of personalized prevention strategies, guiding lifestyle modifications, work schedule adjustments, and other interventions aimed at reducing CKM risk and promoting long-term metabolic health.

### Potential mechanisms

Night shift work and the traits in our study that we used to define circadian imbalance are likely to share serval potential underlying mechanisms involved in CKM disease risk. To date, exposure to night shift work remains the most common and extreme observational model of circadian misalignment in human studies [60], and, similar to circadian imbalance traits, is typically chronic in nature. Night shift work has been associated with an increase in inflammatory markers e.g., level of C-reactive protein (CRP), tumor necrosis factor (TNF-α), and interleukin-6 (IL-6) levels [61], which in turn increase risk of chronic inflammatory conditions such as type 2 diabetes [62] and obesity [63]. Circadian disruption has further been associated with hormonal changes in appetite regulation, including reduced leptin and elevated ghrelin levels, which contribute to weight gain and metabolic dysregulation [64, 65]. On a molecular level, virtually all mammalian cell types have a functional circadian clock including clock and period genes, such as *CLOCK*, *BMAL1 PER1*, *PER2*, *PER3*, *CRY1*, and *CRY2* [66, 67]. Oscillation and dysregulated expression of the molecular circadian clock has been linked to atherosclerosis, insulin resistance, dampening of blood pressure rhythmicity, and reduced production of vasoactive hormones and neurotransmitters [68, 69]. Experimental evidence shows that mice with *CLOCK* gene mutations exhibited disrupted feeding and activity rhythms under ad libitum conditions, leading to obesity and metabolic syndrome [70]. These studies point to the potential mediating effects of obesity, highlighting the importance of our modeling strategy, adding BMI separately in model 4.

Caffeine consumption and bright light/vitamin D have been linked to melatonin secretion and circadian rhythms [8, 71]. Sleep-wake cycle disturbances and exposure to irregular light-dark patterns, as commonly seen in night shift work, may further impair synthesis of cortisol and melatonin [72, 73]. Previous studies consistently support melatonin’s anti-inflammatory, antihypertensive, and oxidative activity and its possibility to reduce the risk of cardiometabolic disease, including type 2 diabetes and hypertension [74, 75]. Therefore, reduced melatonin levels resulting from chronic circadian imbalance or night shift work maybe represent another underlying pathway for the observed associations in our study. Lastly, some lifestyle behaviors such as smoking, sedentary behavior, and irregular meal timing could also be potential contributors to CKM disease. Further studies are needed to explore the pathophysiological pathways underlying the interaction between night shift work and circadian imbalance related traits on CKM disease risk.

In summary, the five components of CII collectively heighten the propensity for circadian desynchrony, and circadian desynchrony can lead to widespread physiological dysregulation [76]. Such potential internal desynchrony may impair the synthesis signaling of melatonin and the synchronized regulation of cortisol and leptin [77]. These hormonal shifts collectively promote systemic inflammation via tumor necrosis factor alpha (TNF-α) and interleukin-6 (IL-6) levels, which ultimately drives systemic metabolic dysfunction [78]. Beyond hormonal shifts, these disruptions trigger cellular-level stress, including mitochondrial dysfunction and the accumulation of reactive oxygen species (ROS) [79].

### Strengths and limitations

The main strengths of this study include its prospective study design, large sample size, and long-term follow up. More importantly, the integration of several circadian imbalance-related traits allowed for a more comprehensive assessment of their potential impact on CKM disease risk. Further, we were able to assess their relationship among different ancestries. Another major novelty of this study is that it is the first to investigate the joint association of night shift work and circadian imbalance related traits with the risk of CKM disease. We also provide novel and unique insight by using detailed shift work metrics including lifetime years and intensity of night shift work. Although uncontrolled confounding remains a limitation in any observational study, the extensive data collection in the UK Biobank allowed for detailed control of potential confounders and mediators.

The present study also has several limitations. First, information on night shift work and most circadian imbalance related factors was self-reported (with the exception of vitamin D which was measured in serum), thus exposure misclassification potentially exists. However, such misclassification would likely be random to outcome status, resulting in attenuation of the effect estimations and underestimation of the observed associations. Further, CII components may act through overlapping behavioral and psychosocial pathways. In addition, information was not available on consumption of caffeinated tea and daily timing of coffee consumption. Furthermore, the CII may not have fully captured all relevant circadian imbalance related traits, such as light exposure or meal timing [80, 81]; both factors are closely linked to circadian timing and are partially reflected by chronotype, which is included in the index. However, in future work, the index could be expanded by incorporating any other relevant factors. Further, although our study leverages the large UK Biobank, the predominance of European ancestry and the healthy volunteer nature of the cohort may limit the generalizability of our findings [82]. Although we observed significant results among Asian participants, these findings may be partly influenced by cultural and socioeconomic contexts specific to Asian populations living in the UK, which may not fully reflect the dynamics of native populations in Asia. In addition, the relatively small sample size of participants of African ancestry limits statistical power, underscoring the need for studies with larger African ancestry populations. Therefore, further validation in diverse, native Asian cohorts and larger African ancestry populations is essential to confirm the external validity of these associations.

### Conclusion and public health implications

In summary, a newly derived Circadian Imbalance Index, CII, integrating evening chronotype, short or long sleep duration, high neuroticism score, atypical caffeine consumption, and low vitamin D levels, was associated with increased CKM disease risk among European and Asian ancestries. Notably, CII and night shift work were jointly associated with a higher risk of CKM disease, and there was an additive association of night shift work and high CII on CKM disease risk. Our findings highlight the possibility that cases of CKM disease could be prevented by reducing high CII scores, which reflect a greater burden of circadian imbalance related traits. The benefits may be particularly pronounced among night shift worker or those with longer duration or greater intensity of night shift exposure. Intervention trials and mechanistic research are warranted to extend this research and clarify the underlying biological mechanisms.

## Supplementary Information

Below is the link to the electronic supplementary material.

Supplementary material 1 (DOCX 675.3 kb)

## Data Availability

The data underlying this article cannot be shared publicly. However, researchers are encouraged to apply to access to the UK Biobank resource for health-related research that serves the public interest. The statistical R code and technical processes are available from the corresponding author.

## Abbreviations

CKM: Cardiovascular-kidney-metabolic
CII: Circadian imbalance index
HRs: Hazard ratios
CIs: Confidence intervals
T2D: Type 2 diabetes
CVD: Cardiovascular disease
CKD: Chronic kidney disease
ICD-10: International classification of diseases
BMI: Body mass index
RERI: Relative excess risk to interaction
AP: Attributable proportion
CRP: C-reactive protein
TNF-α: Tumor necrosis factor
IL-6: Interleukin-6 levels
