# Supplementary material for: A newly developed circadian imbalance index (CII) and risk of cardiovascular-kidney-metabolic disease in the UK biobank
Source: Eur J Epidemiol. 2026 Feb 21;41(4):445–58. doi: 10.1007/s10654-026-01373-7 (PMC13331830; doi:10.1007/s10654-026-01373-7)
Supplement: Supplementary file 1 — Supplementary material 1 (DOCX 675.3 kb) [file 10654_2026_1373_MOESM1_ESM.docx]

Contents

**[Genetic Ancestry Assessment](#_Toc219318560)** [3](#_Toc219318560)

[**Supplementary Statistical Methods** 3](#_Toc219318561)

[**Sensitivity analyses for the association between CII and CKM disease risk** 4](#_Toc219318562)

[**Supplemental Figure 1.** Study flow diagram. 5](#_Toc219318563)

[**Supplemental Figure 2.** Schoenfeld Residuals Test for Proportional Hazards Assumption. 6](#_Toc219318564)

[**Supplemental Figure 3.** Proportion heat map of five negative circadian traits in the Circadian Imbalance Index (CII). 7](#_Toc219318565)

[**Supplemental Figure 4.** Multivariable adjusted hazard ratios, 95% confidence intervals of Cardiovascular-Kidney-Metabolic disease according to joint categories of Circadian Imbalance Index (CII) and other night shift work measurements for European ancestry participants with lifetime occupational questionnaire, n = 47, 843 8](#_Toc219318566)

[**Supplemental Table 1.** UK Biobank Data-Field numbers of covariates used in this study 9](#_Toc219318567)

[**Supplemental Table 2.** Prospective associations between Circadian Imbalance Index (CII) and risk of incident Cardiovascular-Kidney-Metabolic disease in models adjusting for additional covariables among UK Biobank participants, stratified by genetic ancestry (n = 191,764). 10](#_Toc219318568)

[**Supplemental Table 3.** Prospective associations between Circadian Imbalance Index (CII) and risk of incident Cardiovascular-Kidney-Metabolic disease for participants from the UK Biobank, excluding individuals with missing information on covariables, stratified by genetic ancestry (n = 149, 965). 12](#_Toc219318569)

[**Supplemental Table 4a.** Prospective associations between Circadian Imbalance Index (CII) and risk of incident Cardiovascular-Kidney-Metabolic disease for UK Biobank participants, excluding those who reported incident CKM disease within the first two years of follow up, stratified by genetic ancestry (n = 188, 081). 14](#_Toc219318570)

[**Supplemental Table 4b.** Prospective associations between Circadian Imbalance Index (CII) and risk of incident Cardiovascular-Kidney-Metabolic disease for UK Biobank participants, excluding those who reported incident CKM disease within the first 5 years of follow up, stratified by genetic ancestry (n=162,442). 16](#_Toc219318571)

[Supplemental Table 5. Prospective associations between Circadian Imbalance Index (CII) and risk of incident Cardiovascular-Kidney-Metabolic disease for overall participants from the UK Biobank by adjusting genetic ancestry, n = 191,764. 18](#_Toc219318572)

[**Supplemental Table 6.** Prospective associations between Circadian Imbalance Index (CII) and risk of incident Cardiovascular-Kidney-Metabolic disease for participants from the UK biobank, stratified by gender (n= 191,764). 19](#_Toc219318573)

[**Supplemental Table 7.** Prospective associations between Circadian Imbalance Index (CII) and risk of incident Cardiovascular-Kidney-Metabolic disease for participants from the UK biobank, stratified by gender-ancestry. (166,194 European participants, 3,481 Asian participants and 2,583 African participants) 20](#_Toc219318574)

[**Supplemental Table 8.** Prospective associations between Circadian Imbalance Index (CII) and risk of incident Cardiovascular-Kidney-Metabolic for European ancestry UK biobank participants who reported the lifetime occupational history, stratified by duration of night shift work (n = 47,843). 22](#_Toc219318575)

[**Supplemental Table 9.** Prospective associations between Circadian Health Index (CII) and risk of incident Cardiovascular-Kidney-Metabolic disease for European ancestry UK biobank participants who reported on the lifetime occupational history, stratified by the intensity of night shift work (n = 47,843). 24](#_Toc219318576)

[**Supplemental Table 10.** Multivariable adjusted HRs with 95% CI and RERI, AP for additive interaction between Circadian Imbalance Index (CII) and lifetime duration years of night shift work for cardiovascular-kidney-metabolic disease risk among UKB participants European ancestry with occupational history, n = 47, 843 26](#_Toc219318577)

[**Supplemental Table 11.** Multivariable adjusted hazard ratios with 95% CI and RERI, AP for additive interaction between Circadian Imbalance Index (CII) and intensity of night shift work for cardiovascular-kidney-metabolic disease risk among UKB participants European ancestry with occupational history, n = 47, 843 27](#_Toc219318578)

[**Supplemental Table 12.** Prospective associations between Circadian Imbalance Index (CII) and risk of incident type 2 diabetes (T2D) for participants from the UK Biobank, stratified by genetic ethnicity 28](#_Toc219318579)

[**Supplemental Table 13.** Prospective associations between Circadian Imbalance Index (CII) and risk of incident chronic cardiovascular diseases (CVD) for participants from the UK Biobank, stratified by genetic ethnicity 30](#_Toc219318580)

[**Supplemental Table 14.** Prospective associations between Circadian Imbalance Index (CII) and risk of incident chronic kidney diseases (CKD) for participants from the UK Biobank, stratified by genetic ethnicity 32](#_Toc219318581)

[**Supplemental Table 15.** Prospective associations between individual circadian related traits being components of Circadian Imbalance Index (CII) and risk of incident Cardiovascular-Kidney-Metabolic disease for European ancestry participants from the UK Biobank, n = 166,194 34](#_Toc219318582)

[**Supplemental Table 16.** Prospective associations between Circadian Imbalance Index (CII) and risk of incident Cardiovascular-Kidney-Metabolic disease: a cause-specific transition analysis stratified by genetic ethnicity in the UK Biobank 35](#_Toc219318583)

[**Supplemental Table 17.** Prospective associations between Circadian Imbalance Index (CII) and risk of incident Cardiovascular-Kidney-Metabolic disease among 2,583 African ancestry participants from the UK Biobank. 36](#_Toc219318584)

[**Reference:** 37](#_Toc219318585)

**Genetic Ancestry Assessment**

"Genetic ancestry" refers to a statistical construct that reflects the genetic variations an individual has inherited from their ancestors. This concept may not always align with the race or ethnicity that an individual self-identifies. Equating genetic ancestry groups with race or ethnicity is inappropriate, as it significantly oversimplifies the intricate nature of human demographic history and the vast diversity of ethnicities and identities across the globe. The pan UKBB team used a pair of well-known reference panels to define the genetic ancestry groups, which included EUR (European), CSA (Central/South Asian), AFR (African), EAS (East Asian), MID (Middle Eastern), and AMR (Admixed American ancestry)[1]. They used a three-stage approach to analyze genetic ancestry.

Specifically, they: 1) assigned ancestry labels for within-group analysis using reference panel meta-data, 2) visualized population structure within subcontinental ancestries alongside reference panel data, and 3) pruned ancestry outliers within assigned population labels. More details on these and related points can be found in the Pan UKBB website[2].

For our analysis, we combined Central/South Asian and East Asian into Asian ancestry, and combined the unidentified genetic ancestry and Middle Eastern and Admixed American Ancestry into other ancestry.

**Supplementary Statistical Methods**

We applied the following formula [3] to decompose the joint excess relative risk for both exposures (HR_11_-1) into the excess relative risk for night shift work alone (HR_01_-1), CII pattern alone (HR_10_-1) and relative excess risk due to interaction (RERI).

$$\mathrm{HR}_{11}-1=\left( \mathrm{HR}_{01}-1 \right)+\left( \mathrm{HR}_{10}-1 \right)+RERI$$

For the AP[3], we likewise calculated the proportion of the joint effect due to shift work alone (HR_01_-1)/(HR_11_-1), due to CII pattern alone (HR_10_-1) /(HR_11_-1), and due to their additive interaction, RERI/(HR_11_-1).

**Sensitivity analyses for the association between CII and CKM disease risk**

In sensitivity analyses, we additionally adjusted our models for hypertension, hyperlipidemia and recruitment season (Supplemental Table 2), excluded all missing covariates (Supplemental Table 3), or CKM cases diagnosed in the first two years of follow up (Supplemental Table 4), and the association patterns were consistent with the main analyses. Due to the decreasing CKM cases and statistical power, we observed the insignificant association for CII of 5 compared the CII of 0-1 in the Asian ancestry at the above three sensitivity analyses (Supplemental Tables 2-4). We further performed analyses between CII and each individual disease composing CKM, (type 2 diabetes (T2D), cardiovascular diseases (CVD), and chronic kidney disease (CKD)). We observed the similar significant increasing risk association between CII and each individual disease (T2D: *P* trend < 0.001, CVD: *P* trend < 0.001, CKD: *P* trend < 0.001) among participants of European ancestry. Among Asian ancestry participants, we only observed the significant *P* trend for the association between CII and T2D. No significant result were observed among participants of African ancestry (Supplemental Tables 11-13). In additional analyses, we converted the five circadian traits into binary traits (positive vs. negative), and calculated the proportion between them obtaining weak to moderate values (Supplemental Figure 3). Then, we examined the associations of individual traits with the risk of CKM disease. Evening chronotype, abnormal sleep duration, a neuroticism score of ≥ 7, atypical caffeinated coffee consumption, and low serum vitamin D level were each independently associated with incident CKM disease. After mutually adjusting for the traits of others and variables listed above in the Model 2, these associations remain statistically significant, with a 5%, 21% 13%, 13%, and 32% higher risk for risk of CKM disease, respectively (Supplemental Table 14).

**Supplemental Figure 1.** Study flow diagram.


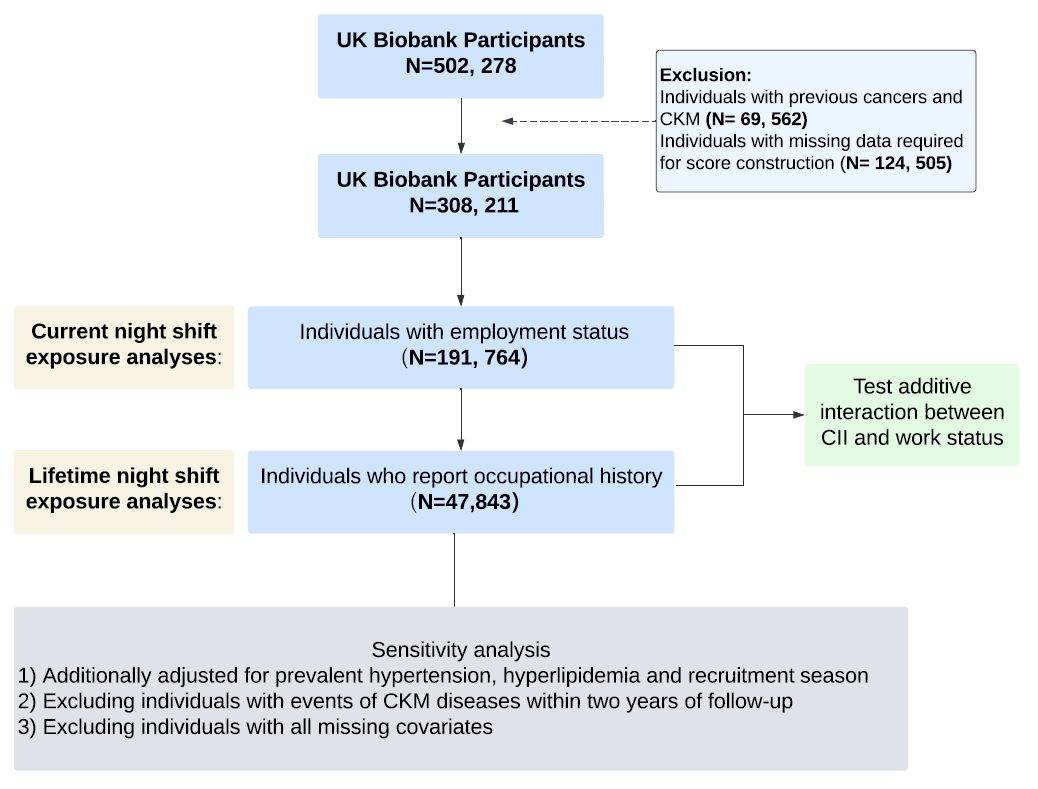


**Supplemental Figure 2.** Schoenfeld Residuals Test for Proportional Hazards Assumption.


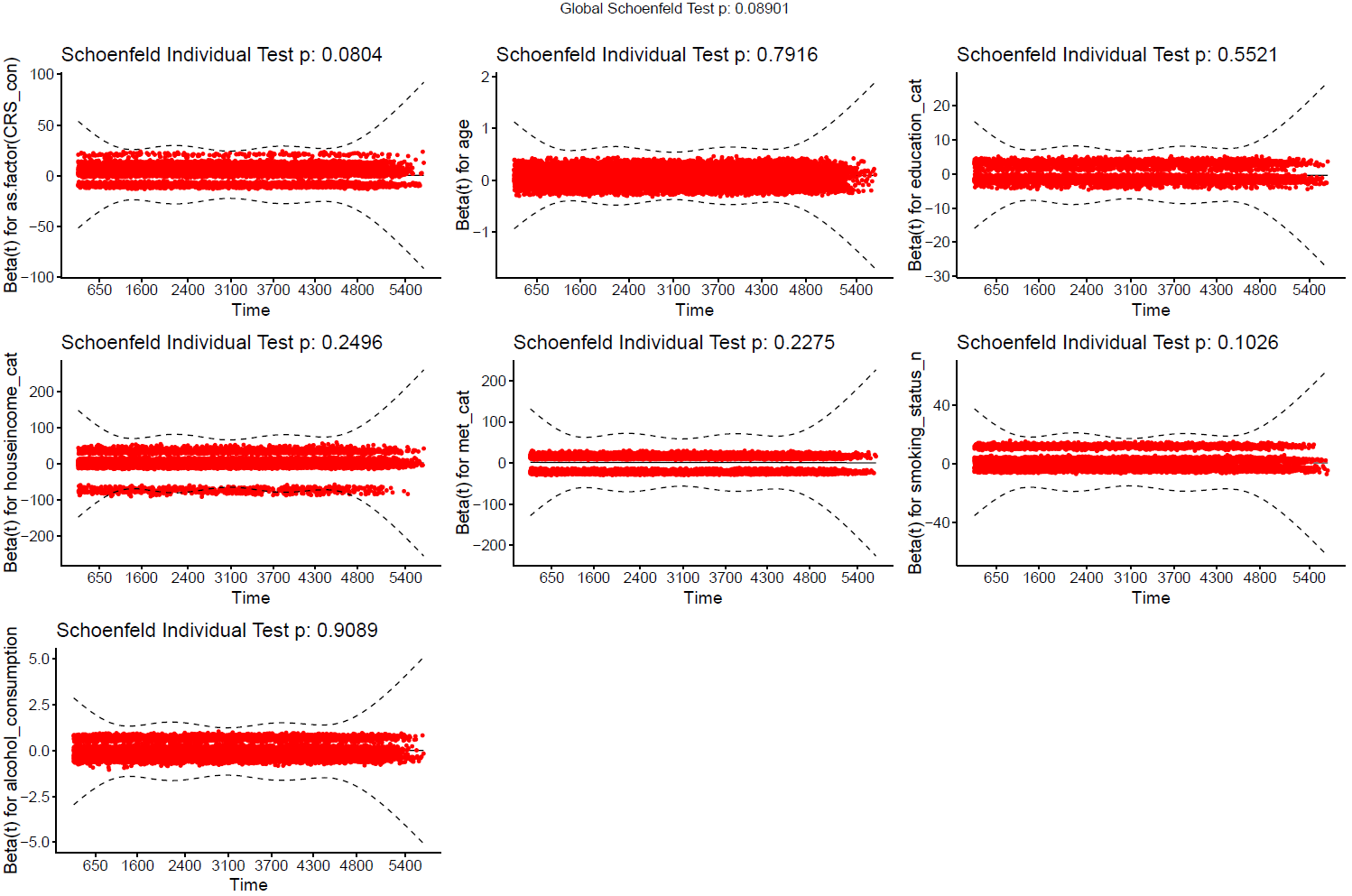


**Supplemental Figure 3.** Proportion heat map of five negative circadian traits in the Circadian Imbalance Index (CII).


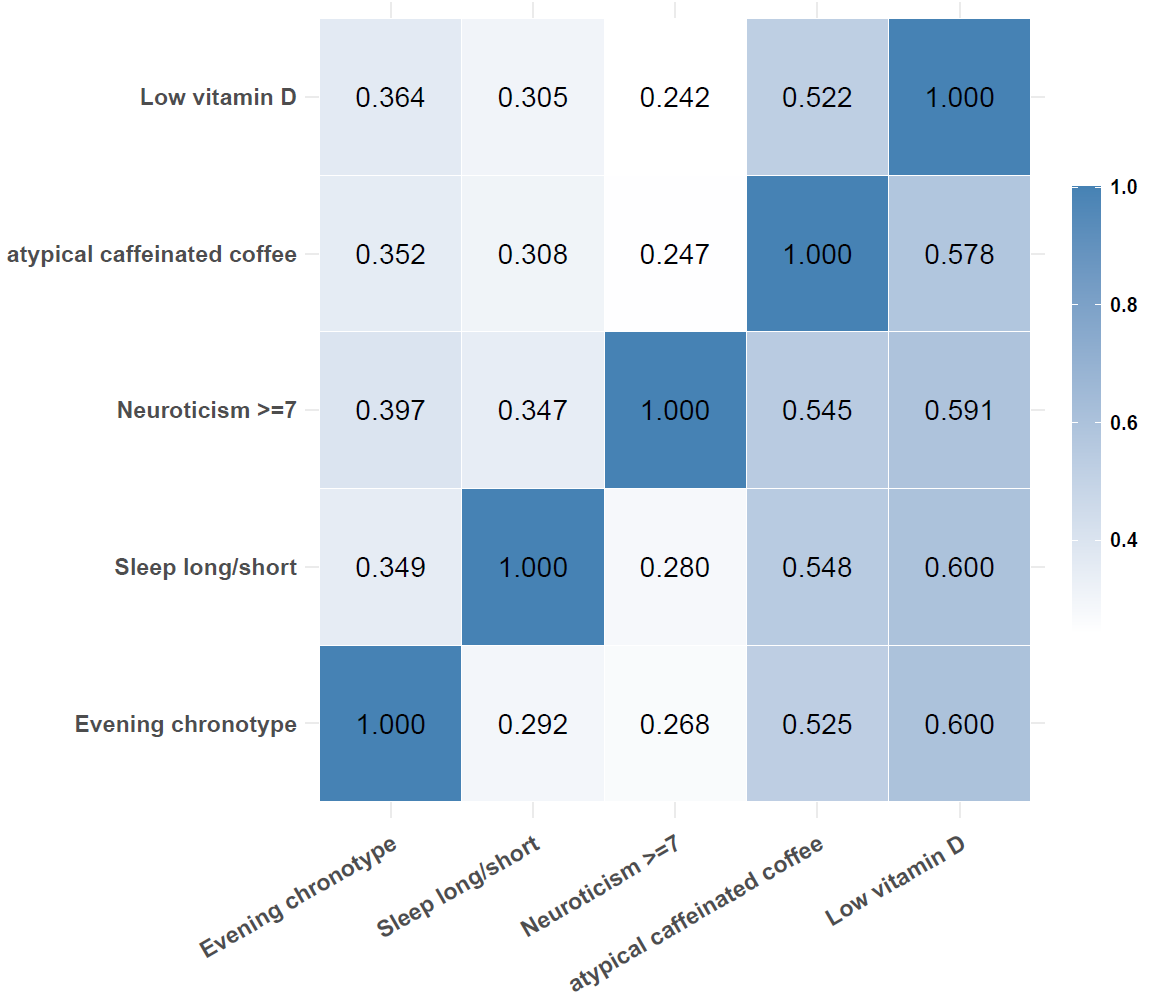


**Supplemental Figure 4.** Multivariable adjusted hazard ratios, 95% confidence intervals of Cardiovascular-Kidney-Metabolic disease according to joint categories of Circadian Imbalance Index (CII) and other night shift work measurements for European ancestry participants with lifetime occupational questionnaire, n = 47, 843


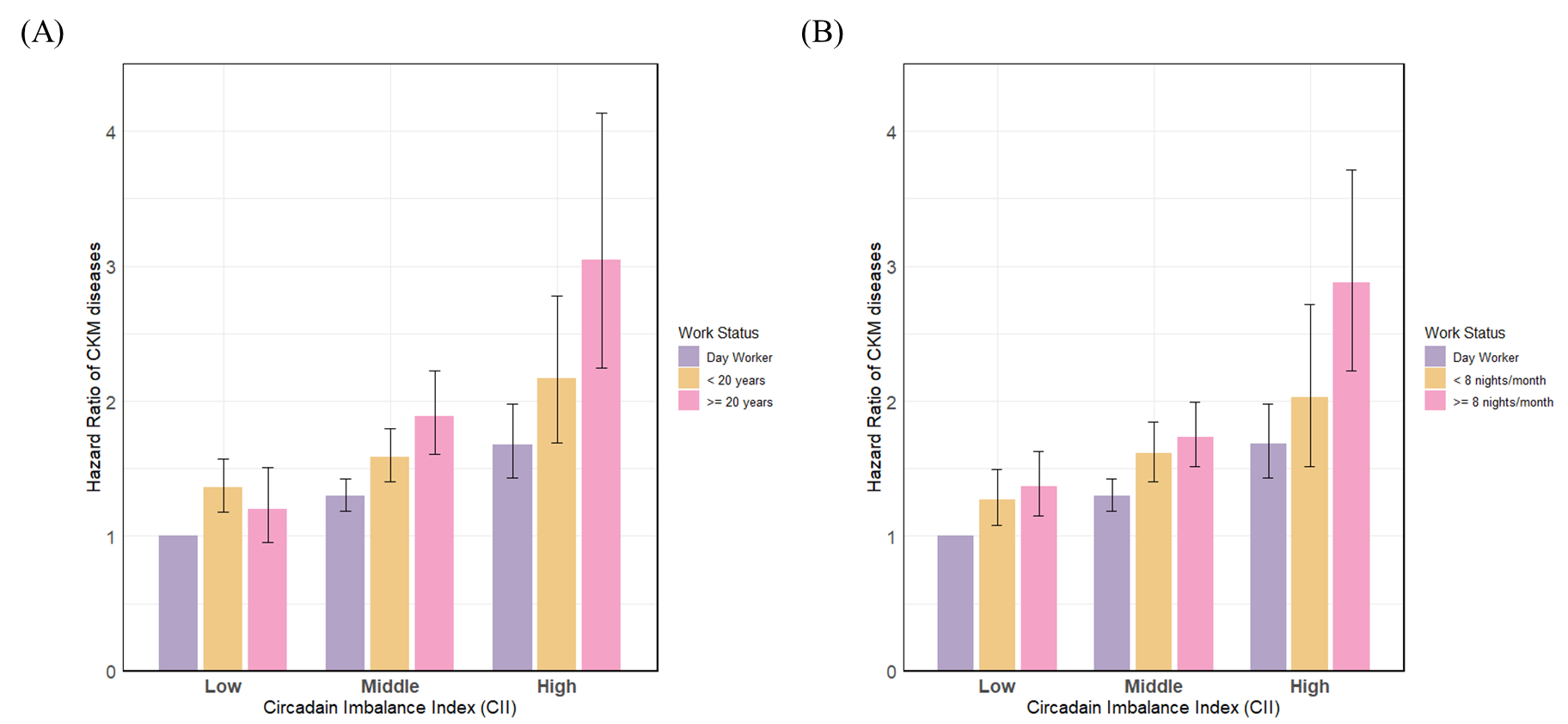


A) Joint association of Circadian Imbalance Index (CII) and duration years of night shift work, B) Joint association of Circadian Imbalance Index (CII) and intensity of night shift work, multivariable adjusted for sex, age, household income and education.

**Supplemental Table 1.** UK Biobank Data-Field numbers of covariates used in this study

| **Variable** | **Data-Field** | **Notes on variable definition** |
| --- | --- | --- |
| Date of attending assessment center | 53 | -- |
| Age | 21003 | Continuous |
| Sex | 31 | Binary |
| Average total household income after tax | 738 | Categorical: ‘<£30.9k’, ‘£30.9k-100k’, ‘>£100k’, and ‘Unknown’ |
| Education | 6138 | Binary: ‘College’ and ‘other non0colleague’ (selection of any education category except ‘College’) |
| Recruitment season | 53 | Categorical: ‘Spring’, ‘Summer’, ‘Autumn’, and ‘Winter’. Deprived from the date of attending assessment center. |
| Hypertension | 4080, 4079 | Binary: ‘high’ = systolic > 140 or diastolic > 90 |
| Elevated cholesterol | 20002 | Elevated cholesterol defined according to the self-reported disease. |
| Smoking status | 20116 | Categorical: ‘Current’, ‘Previous’, ‘Never’ and ‘Unknown’ |
| Alcohol assumption | 1558 | Continuous, days per week consuming alcohol. ‘Daily’ = 7, ‘3-4 times per week’ = 3·5, ‘1-2 times per week’ = 1·5, ‘1-3 times per month’ = 0.46, ‘special occasions only’ = 0.23, ‘never’ = 0 |
| Physical activity | 22040 | Categorical: ‘<10 Total Metabolic Equivalent Task (MET) minutes per week for all activity including walking, moderate and vigorous activity’, ’10-50 MET-h/week’, ‘>50 MET-h/week’ and ‘Unknown’ |
| Body mass index (BMI) | 21001 | Continuous |

**Supplemental Table 2.** Prospective associations between Circadian Imbalance Index (CII) and risk of incident Cardiovascular-Kidney-Metabolic disease in models adjusting for additional covariables among UK Biobank participants, stratified by genetic ancestry (n = 191,764).

| **Ethnicity** | **CII** | **Case/N** | **Model 1^a^** | | **Model 4^b^** | | **Model 5^c^** | | **Model 6^d^** | |
| --- | --- | --- | --- | --- | --- | --- | --- | --- | --- | --- |
|  |  |  | **HR (95%CI)** | ***P*_trend_** | **HR(95%CI)** | ***P*_trend_** | **HR(95%CI)** | ***P*_trend_** | **HR(95%CI)** | ***P*_trend_** |
| **European (N=166,194)** | 0-1 | 4 474/59 921 | Reference | <0.001 | Reference | <0.001 | Reference | <0.001 | Reference | <0.001 |
|  | 2 | 4 979/56 698 | 1.27 (1.22-1.32) |  | 1.14 (1.09-1.18) |  | 1.13 (1.09-1.18) |  | 1.15 (1.10-1.19) |  |
|  | 3 | 3 394/35 600 | 1.47 (1.40-1.53) |  | 1.21 (1.16-1.27) |  | 1.21 (1.15-1.26) |  | 1.23 (1.17-1.28) |  |
|  | 4 | 1 269/12 058 | 1.74 (1.63-1.85) |  | 1.34 (1.26-1.42) |  | 1.33 (1.24-1.41) |  | 1.35 (1.26-1.44) |  |
|  | 5 | 221/ 1 917 | 2.08 (1.82-2.38) |  | 1.42 (1.24-1.63) |  | 1.40 (1.22-1.60) |  | 1.42 (1.24-1.63) |  |
| **Asian (N=3,481)** | 0-1 | 67/ 562 | Reference | <0.001 | Reference | 0.024 | Reference | 0.019 | Reference | 0.020 |
|  | 2 | 211/1 350 | 1.42 (1.08-1.87) |  | 1.37 (1.04-1.80) |  | 1.34 (1.02-1.77) |  | 1.34 (1.02-1.77) |  |
|  | 3 | 183/1 103 | 1.51 (1.14-2.00) |  | 1.36 (1.03-1.81) |  | 1.38 (1.04-1.84) |  | 1.39 (1.04-1.84) |  |
|  | 4 | 68/ 398 | 1.71 (1.22-2.39) |  | 1.49 (1.06-2.09) |  | 1.47 (1.04-2.07) |  | 1.47 (1.04-2.07) |  |
|  | 5 | 11/ 68 | 2.13 (1.12-4.05) |  | 1.82 (0.95-3.48) |  | 1.82 (0.95-3.50) |  | 1.80 (0.94-3.46) |  |
| **African (N=2,583)** | 0-1 | 42/333 | Reference | 0.326 | Reference | 0.662 | Reference | 0.608 | Reference | 0.510 |
|  | 2 | 141/897 | 1.39 (0.99-1.96) |  | 1.29 (0.91-1.82) |  | 1.34 (0.95-1.90) |  | 1.35 (0.95-1.90) |  |
|  | 3 | 142/946 | 1.32 (0.94-1.86) |  | 1.26 (0.89-1.78) |  | 1.27 (0.90-1.80) |  | 1.29 (0.91-1.82) |  |
|  | 4 | 52/349 | 1.32 (0.88-1.98) |  | 1.16 (0.77-1.74) |  | 1.22 (0.81-1.84) |  | 1.24 (0.82-1.87) |  |
|  | 5 | 8/ 58 | 1.46 (0.68-3.11) |  | 1.24 (0.58-2.65) |  | 1.22 (0.57-2.62) |  | 1.27 (0.59-2.74) |  |
| ^a^Model 1 includes sex and age | | | | | | | | | | |
| ^b^Model 4 includes variables in Model 1 and household income, education, smoking status, alcohol consumption, physical activity and body mass index (BMI) | | | | | | | | | | |
| ^c^Model 5 includes variables in Model 4 and hypertension and hyperlipidemia | | | | | | | | | | |
| ^d^Model 6 includes variables in Model 4 and recruitment season | | | | | | | | | | |

**Supplemental Table 3.** Prospective associations between Circadian Imbalance Index (CII) and risk of incident Cardiovascular-Kidney-Metabolic disease for participants from the UK Biobank, excluding individuals with missing information on covariables, stratified by genetic ancestry (n = 149, 965).

| **Ethnicity** | **CII** | **Case/N** | **Model 1^a^** | | **Model 2^b^** | | **Model 3^c^** | | **Model 4^d^** | |
| --- | --- | --- | --- | --- | --- | --- | --- | --- | --- | --- |
|  |  |  | **HR (95%CI)** | ***P*_trend_** | **HR (95%CI)** | ***P*_trend_** | **HR (95%CI)** | ***P*_trend_** | **HR (95%CI)** | ***P*_trend_** |
| **European (N=130,390)** | 0-1 | 3 406/47 831 | Reference | <0.001 | Reference | <0.001 | Reference | <0.001 | Reference | <0.001 |
|  | 2 | 3 713/44 506 | 1.26 (1.21-1.32) |  | 1.25 (1.19-1.31) |  | 1.21 (1.15-1.27) |  | 1.13 (1.08-1.18) |  |
|  | 3 | 2 480/27 556 | 1.45 (1.38-1.53) |  | 1.41 (1.33-1.48) |  | 1.33 (1.27-1.41) |  | 1.20 (1.14-1.27) |  |
|  | 4 | 923/ 9 062 | 1.75 (1.62-1.88) |  | 1.66 (1.54-1.79) |  | 1.55 (1.44-1.67) |  | 1.34 (1.24-1.44) |  |
|  | 5 | 153/ 1 435 | 1.98 (1.69-2.33) |  | 1.85 (1.58-2.18) |  | 1.68 (1.42-1.97) |  | 1.32 (1.12-1.56) |  |
| **Asian (N=2,569)** | 0-1 | 46/ 429 | Reference | 0.005 | Reference | 0.011 | Reference | 0.021 | Reference | 0.067 |
|  | 2 | 149/1 011 | 1.49 (1.07-2.07) |  | 1.48 (1.07-2.07) |  | 1.45 (1.04-2.03) |  | 1.41 (1.01-1.97) |  |
|  | 3 | 125/ 801 | 1.58 (1.12-2.21) |  | 1.57 (1.12-2.20) |  | 1.53 (1.09-2.15) |  | 1.41 (1.00-1.98) |  |
|  | 4 | 48/ 284 | 1.86 (1.24-2.80) |  | 1.78 (1.19-2.68) |  | 1.70 (1.13-2.56) |  | 1.59 (1.06-2.40) |  |
|  | 5 | 4/ 44 | 1.38 (0.50-3.87) |  | 1.31 (0.47-3.65) |  | 1.25 (0.45-3.51) |  | 1.22 (0.44-3.43) |  |
| **African (N=1,807)** | 0-1 | 30/244 | Reference | 0.193 | Reference | 0.206 | Reference | 0.258 | Reference | 0.347 |
|  | 2 | 90/620 | 1.33 (0.88-2.01) |  | 1.32 (0.87-1.99) |  | 1.28 (0.84-1.94) |  | 1.28 (0.85-1.94) |  |
|  | 3 | 95/662 | 1.31 (0.87-1.98) |  | 1.30 (0.86-1.96) |  | 1.26 (0.83-1.90) |  | 1.29 (0.85-1.94) |  |
|  | 4 | 40/242 | 1.48 (0.92-2.38) |  | 1.48 (0.92-2.37) |  | 1.45 (0.90-2.33) |  | 1.37 (0.85-2.21) |  |
|  | 5 | 5/ 39 | 1.29 (0.50-3.32) |  | 1.26 (0.49-3.25) |  | 1.16 (0.45-3.01) |  | 1.07 (0.41-2.79) |  |
| ^a^Model 1 includes sex and age | | | | | | | | | | |
| ^b^Model 2 includes variables in Model 1 and household income and education | | | | | | | | | | |
| ^c^Model 3 includes variables in Model 2 and smoking status, alcohol consumption and physical activity | | | | | | | | | | |
| ^d^Model 4 includes variables in Model 3 and body mass index (BMI) | | | | | | | | | | |

**Supplemental Table 4a.** Prospective associations between Circadian Imbalance Index (CII) and risk of incident Cardiovascular-Kidney-Metabolic disease for UK Biobank participants, excluding those who reported incident CKM disease within the first two years of follow up, stratified by genetic ancestry (n = 188, 081).

| **Ethnicity** | **CII** | **Case/N** | **Model 1^a^** | | **Model 2^b^** | | **Model 3^c^** | | **Model 4^d^** | |
| --- | --- | --- | --- | --- | --- | --- | --- | --- | --- | --- |
|  |  |  | **HR (95%CI)** | ***P*_trend_** | **HR (95%CI)** | ***P*_trend_** | **HR (95%CI)** | ***P*_trend_** | **HR (95%CI)** | ***P*_trend_** |
| **European**  **(N=163, 040)** | 0-1 | 4 124/58 850 | Reference | <0.001 | Reference | <0.001 | Reference | <0.001 | Reference | <0.001 |
|  | 2 | 4 537/55 616 | 1.26 (1.21-1.31) |  | 1.24 (1.19-1.29) |  | 1.20 (1.15-1.26) |  | 1.12 (1.08-1.17) |  |
|  | 3 | 3 074/34 874 | 1.44 (1.37-1.51) |  | 1.40 (1.33-1.46) |  | 1.32 (1.26-1.38) |  | 1.19 (1.14-1.25) |  |
|  | 4 | 1 162/11 823 | 1.72 (1.61-1.84) |  | 1.64 (1.54-1.75) |  | 1.52 (1.43-1.63) |  | 1.33 (1.25-1.42) |  |
|  | 5 | 204/ 1 877 | 2.08 (1.81-2.40) |  | 1.95 (1.70-2.25) |  | 1.76 (1.53-2.02) |  | 1.43 (1.24-1.65) |  |
| **Asian**  **(N=2,512)** | 0-1 | 59/ 550 | Reference | 0.002 | Reference | 0.004 | Reference | 0.012 | Reference | 0.039 |
|  | 2 | 190/1 322 | 1.46 (1.09-1.95) |  | 1.44 (1.08-1.93) |  | 1.42 (1.06-1.91) |  | 1.40 (1.04-1.87) |  |
|  | 3 | 164/1 078 | 1.54 (1.14-2.07) |  | 1.52 (1.13-2.04) |  | 1.46 (1.08-1.97) |  | 1.39 (1.03-1.87) |  |
|  | 4 | 64/ 393 | 1.83 (1.28-2.60) |  | 1.76 (1.23-2.51) |  | 1.68 (1.17-2.40) |  | 1.58 (1.11-2.26) |  |
|  | 5 | 7/ 64 | 1.52 (0.69-3.34) |  | 1.44 (0.65-3.17) |  | 1.39 (0.63-3.07) |  | 1.31 (0.59-2.89) |  |
| **African**  **(N=3,407)** | 0-1 | 37/323 | Reference | 0.428 | Reference | 0.468 | Reference | 0.580 | Reference | 0.824 |
|  | 2 | 127/877 | 1.43 (0.99-2.06) |  | 1.42 (0.98-2.04) |  | 1.35 (0.94-1.96) |  | 1.32 (0.91-1.90) |  |
|  | 3 | 125/916 | 1.32 (0.91-1.90) |  | 1.29 (0.90-1.87) |  | 1.24 (0.86-1.80) |  | 1.25 (0.86-1.81) |  |
|  | 4 | 46/340 | 1.31 (0.85-2.03) |  | 1.30 (0.84-2.01) |  | 1.25 (0.81-1.93) |  | 1.14 (0.74-1.76) |  |
|  | 5 | 7/ 56 | 1.45 (0.64-3.26) |  | 1.42 (0.63-3.20) |  | 1.35 (0.60-3.06) |  | 1.22 (0.54-2.75) |  |
| ^a^Model 1 includes sex and age | | | | | | | | | | |
| ^b^Model 2 includes variables in Model 1 and household income and education | | | | | | | | | | |
| ^c^Model 3 includes variables in Model 2 and smoking status, alcohol consumption and physical activity | | | | | | | | | | |
| ^d^Model 4 includes variables in Model 3 and body mass index (BMI) | | | | | | | | | | |

**Supplemental Table 4b.** Prospective associations between Circadian Imbalance Index (CII) and risk of incident Cardiovascular-Kidney-Metabolic disease for UK Biobank participants, excluding those who reported incident CKM disease within the first 5 years of follow up, stratified by genetic ancestry (n=162,442).

| **Ethnicity** | **CII** | **Case/N** | **Model 1^a^** | | **Model 2^b^** | | **Model 3^c^** | | **Model 4^d^** | |
| --- | --- | --- | --- | --- | --- | --- | --- | --- | --- | --- |
|  |  |  | **HR (95%CI)** | ***P*_trend_** | **HR (95%CI)** | ***P*_trend_** | **HR (95%CI)** | ***P*_trend_** | **HR (95%CI)** | ***P*_trend_** |
| **European**  **(N= 156,779)** | 0-1 | 3 344/56 671 | Reference | <0.001 | Reference | <0.001 | Reference | <0.001 | Reference | <0.001 |
|  | 2 | 3 673/53 465 | 1.26(1.20-1.32) |  | 1.24(1.18-1.30) |  | 1.20(1.15-1.26) |  | 1.13(1.07-1.18) |  |
|  | 3 | 2 514/33 520 | 1.45(1.38-1.53) |  | 1.41(1.33-1.48) |  | 1.33(1.27-1.41) |  | 1.21(1.15-1.27) |  |
|  | 4 | 934/11 327 | 1.70(1.59-1.83) |  | 1.62(1.51-1.74) |  | 1.51(1.40-1.62) |  | 1.32(1.23-1.43) |  |
|  | 5 | 163/ 1 796 | 2.05(1.75-2.40) |  | 1.92(1.64-2.25) |  | 1.73(1.48-2.03) |  | 1.42(1.21-1.67) |  |
| **Asian**  **(N= 3,260)** | 0-1 | 51/ 534 | Reference | 0.037 | Reference | 0.066 | Reference | 0.106 | Reference | 0.216 |
|  | 2 | 154/1 265 | 1.37(1.00-1.89) |  | 1.36(0.99-1.87) |  | 1.35(0.98-1.85) |  | 1.33(0.97-1.82) |  |
|  | 3 | 127/1 026 | 1.39(1.00-1.92) |  | 1.36(0.98-1.88) |  | 1.32(0.95-1.83) |  | 1.26(0.91-1.75) |  |
|  | 4 | 49/ 373 | 1.64(1.10-2.43) |  | 1.57(1.06-2.32) |  | 1.52(1.02-2.25) |  | 1.44(0.97-2.14) |  |
|  | 5 | 5/ 62 | 1.26(0.50-3.18) |  | 1.19(0.47-3.00) |  | 1.17(0.46-2.95) |  | 1.10(0.43-2.78) |  |
| **African**  **(N= 2,403)** | 0-1 | 29/313 | Reference | 0.334 | Reference | 0.379 | Reference | 0.463 | Reference | 0.667 |
|  | 2 | 100/829 | 1.43(0.95-2.17) |  | 1.43(0.94-2.16) |  | 1.37(0.90-2.07) |  | 1.33(0.88-2.01) |  |
|  | 3 | 103/882 | 1.37(0.91-2.07) |  | 1.34(0.89-2.03) |  | 1.29(0.86-1.96) |  | 1.29(0.86-1.96) |  |
|  | 4 | 36/326 | 1.29(0.79-2.11) |  | 1.28(0.78-2.08) |  | 1.23(0.75-2.00) |  | 1.12(0.69-1.84) |  |
|  | 5 | 7/ 53 | 1.84(0.80-4.21) |  | 1.82(0.79-4.16) |  | 1.73(0.75-3.97) |  | 1.54(0.67-3.56) |  |
| ^a^Model 1 includes sex and age | | | | | | | | | | |
| ^b^Model 2 includes variables in Model 1 and household income and education | | | | | | | | | | |
| ^c^Model 3 includes variables in Model 2 and smoking status, alcohol consumption and physical activity | | | | | | | | | | |
| ^d^Model 4 includes variables in Model 3 and body mass index (BMI) | | | | | | | | | | |

Supplemental Table 5. Prospective associations between Circadian Imbalance Index (CII) and risk of incident Cardiovascular-Kidney-Metabolic disease for overall participants from the UK Biobank by adjusting genetic ancestry, n = 191,764.

| **CII** | **Case/N** | **Model 1^a^** | | **Model 2^b^** | | **Model 3^c^** | | **Model 4^d^** | |
| --- | --- | --- | --- | --- | --- | --- | --- | --- | --- |
|  |  | **HR (95%CI)** | ***P*_trend_** | **HR (95%CI)** | ***P*_trend_** | **HR (95%CI)** | ***P*_trend_** | **HR (95%CI)** | ***P*_trend_** |
| 0-1 | 5 037/66 962 | Reference | <0.001 | Reference | <0.001 | Reference | <0.001 | Reference | <0.001 |
| 2 | 5 919/65 765 | 1.30 (1.25-1.34) |  | 1.28 (1.23-1.33) |  | 1.23 (1.19-1.28) |  | 1.16 (1.12-1.20) |  |
| 3 | 4 136/42 246 | 1.50 (1.44-1.56) |  | 1.45 (1.39-1.51) |  | 1.37 (1.31-1.43) |  | 1.24 (1.19-1.29) |  |
| 4 | 1 550/14 468 | 1.76 (1.66-1.86) |  | 1.67 (1.58-1.77) |  | 1.54 (1.46-1.63) |  | 1.36 (1.28-1.44) |  |
| 5 | 265/ 2 323 | 2.06 (1.82-2.33) |  | 1.93 (1.71-2.19) |  | 1.73 (1.53-1.96) |  | 1.42 (1.26-1.61) |  |
| ^a^Model 1 includes sex, age and genetic ancestry | | | | | | | | | |
| ^b^Model 2 includes variables in Model 1 and household income and education | | | | | | | | | |
| ^c^Model 3 includes variables in Model 2 and smoking status, alcohol consumption and physical activity | | | | | | | | | |
| ^d^Model 4 includes variables in Model 3 and body mass index (BMI) | | | | | | | | | |

**Supplemental Table 6.** Prospective associations between Circadian Imbalance Index (CII) and risk of incident Cardiovascular-Kidney-Metabolic disease for participants from the UK biobank, stratified by gender (n= 191,764).

| **Group** | **CII** | **Case/N** | **Model 1^a^** | | **Model 2^b^** | | **Model 3^c^** | | **Model 4^d^** | |
| --- | --- | --- | --- | --- | --- | --- | --- | --- | --- | --- |
|  |  |  | **HR (95%CI)** | ***P*_trend_** | **HR (95%CI)** | ***P*_trend_** | **HR (95%CI)** | ***P*_trend_** | **HR (95%CI)** | ***P*_trend_** |
| **Women (N = 97,490)** | 0-1 | 1 558/32 271 | Reference | <0.001 | Reference | <0.001 | Reference | <0.001 | Reference | <0.001 |
|  | 2 | 2 049/33 506 | 1.29 (1.21-1.38) |  | 1.27 (1.19-1.36) |  | 1.22 (1.15-1.31) |  | 1.12 (1.05-1.20) |  |
|  | 3 | 1 564/22 350 | 1.51 (1.40-1.62) |  | 1.45 (1.36-1.56) |  | 1.36 (1.27-1.46) |  | 1.20 (1.12-1.29) |  |
|  | 4 | 635/ 8 023 | 1.77 (1.61-1.94) |  | 1.67 (1.52-1.83) |  | 1.51 (1.38-1.66) |  | 1.27 (1.16-1.39) |  |
|  | 5 | 131/ 1 340 | 2.27 (1.90-2.71) |  | 2.11 (1.76-2.52) |  | 1.86 (1.55-2.22) |  | 1.43 (1.19-1.71) |  |
| **Men (N = 94,274)** | 0-1 | 3 479/34 691 | Reference | <0.001 | Reference | <0.001 | Reference | <0.001 | Reference | <0.001 |
|  | 2 | 3 870/32 259 | 1.26 (1.21-1.32) |  | 1.25 (1.20-1.31) |  | 1.21 (1.16-1.27) |  | 1.15 (1.10-1.20) |  |
|  | 3 | 2 572/19 896 | 1.42 (1.35-1.50) |  | 1.39 (1.32-1.46) |  | 1.32 (1.25-1.39) |  | 1.21 (1.15-1.28) |  |
|  | 4 | 915/ 6 445 | 1.66 (1.54-1.79) |  | 1.59 (1.48-1.72) |  | 1.49 (1.39-1.61) |  | 1.35 (1.25-1.45) |  |
|  | 5 | 134/ 983 | 1.81 (1.52-2.15) |  | 1.71 (1.44-2.03) |  | 1.56 (1.31-1.85) |  | 1.35 (1.14-1.61) |  |
| ^a^Model 1 includes age and genetic ancestry | | | | | | | | | | |
| ^b^Model 2 includes variables in Model 1 and household income and education | | | | | | | | | | |
| ^c^Model 3 includes variables in Model 2 and smoking status, alcohol consumption and physical activity | | | | | | | | | | |
| ^d^Model 4 includes variables in Model 3 and body mass index (BMI) | | | | | | | | | | |

**Supplemental Table 7.** Prospective associations between Circadian Imbalance Index (CII) and risk of incident Cardiovascular-Kidney-Metabolic disease for participants from the UK biobank, stratified by gender-ancestry. (166,194 European participants, 3,481 Asian participants and 2,583 African participants)

| **Group** | **CII** | **Case/N** | **Model 1^a^** | | **Model 2^b^** | | **Model 3^c^** | | **Model 4^d^** | |
| --- | --- | --- | --- | --- | --- | --- | --- | --- | --- | --- |
|  |  |  | **HR (95%CI)** | ***P*_trend_** | **HR (95%CI)** | ***P*_trend_** | **HR (95%CI)** | ***P*_trend_** | **HR (95%CI)** | ***P*_trend_** |
| **European Women**  **(N=84,045)** | 0-1 | 1 344/28 677 | Reference | <0.001 | Reference | <0.001 | Reference | <0.001 | Reference | <0.001 |
|  | 2 | 1 690/28 783 | 1.31 (1.22-1.40) |  | 1.29 (1.20-1.38) |  | 1.24 (1.15-1.33) |  | 1.14 (1.06-1.22) |  |
|  | 3 | 1 257/18 822 | 1.53 (1.42-1.65) |  | 1.48 (1.37-1.60) |  | 1.38 (1.28-1.49) |  | 1.21 (1.12-1.31) |  |
|  | 4 | 514/ 6 685 | 1.83 (1.66-2.03) |  | 1.73 (1.56-1.92) |  | 1.56 (1.41-1.73) |  | 1.31 (1.18-1.45) |  |
|  | 5 | 106/ 1 078 | 2.43 (1.99-2.96) |  | 2.27 (1.86-2.76) |  | 1.97 (1.61-2.40) |  | 1.50 (1.23-1.83) |  |
| **European Men**  **(N=82,149)** | 0-1 | 3 130/31 244 | Reference | <0.001 | Reference | <0.001 | Reference | <0.001 | Reference | <0.001 |
|  | 2 | 3 289/27 915 | 1.25 (1.19-1.32) |  | 1.24 (1.18-1.30) |  | 1.20 (1.15-1.26) |  | 1.13 (1.08-1.19) |  |
|  | 3 | 2 137/16 778 | 1.43 (1.36-1.51) |  | 1.39 (1.32-1.47) |  | 1.32 (1.25-1.40) |  | 1.21 (1.14-1.28) |  |
|  | 4 | 755/ 5 373 | 1.68 (1.55-1.82) |  | 1.61 (1.49-1.74) |  | 1.51 (1.39-1.63) |  | 1.36 (1.25-1.47) |  |
|  | 5 | 115/ 839 | 1.84 (1.53-2.22) |  | 1.73 (1.44-2.09) |  | 1.58 (1.31-1.90) |  | 1.34 (1.11-1.62) |  |
| **Asian Women**  **(N=1,622)** | 0-1 | 24/262 | Reference | 0.245 | Reference | 0.328 | Reference | 0.478 | Reference | 0.995 |
|  | 2 | 69/631 | 1.18 (0.74-1.89) |  | 1.14 (0.72-1.82) |  | 1.10 (0.69-1.76) |  | 1.08 (0.68-1.73) |  |
|  | 3 | 56/496 | 1.29 (0.80-2.08) |  | 1.22 (0.76-1.98) |  | 1.17 (0.72-1.90) |  | 1.03 (0.63-1.67) |  |
|  | 4 | 21/188 | 1.31 (0.73-2.35) |  | 1.28 (0.71-2.29) |  | 1.18 (0.65-2.13) |  | 1.04 (0.57-1.89) |  |
|  | 5 | 5/ 45 | 1.53 (0.58-4.02) |  | 1.36 (0.52-3.60) |  | 1.28 (0.48-3.39) |  | 1.07 (0.40-2.86) |  |
| **Asian Men**  **(N=1,859)** | 0-1 | 43/300 | Reference | <0.001 | Reference | 0.002 | Reference | 0.004 | Reference | 0.006 |
|  | 2 | 142/719 | 1.55 (1.10-2.18) |  | 1.55 (1.10-2.19) |  | 1.54 (1.09-2.17) |  | 1.53 (1.08-2.15) |  |
|  | 3 | 127/607 | 1.63 (1.16-2.31) |  | 1.64 (1.16-2.32) |  | 1.58 (1.12-2.24) |  | 1.55 (1.10-2.20) |  |
|  | 4 | 47/210 | 1.94 (1.28-2.93) |  | 1.88 (1.24-2.86) |  | 1.83 (1.20-2.78) |  | 1.76 (1.16-2.69) |  |
|  | 5 | 6/ 23 | 2.72 (1.15-6.43) |  | 2.59 (1.10-6.13) |  | 2.57 (1.08-6.15) |  | 2.72 (1.14-6.51) |  |
| **African Women**  **(N=1,481)** | 0-1 | 25/182 | Reference | 0.508 | Reference | 0.530 | Reference | 0.776 | Reference | 0.857 |
|  | 2 | 77/513 | 1.24 (0.79-1.94) |  | 1.22 (0.78-1.93) |  | 1.08 (0.69-1.71) |  | 1.08 (0.69-1.71) |  |
|  | 3 | 74/531 | 1.18 (0.75-1.86) |  | 1.17 (0.75-1.85) |  | 1.06 (0.67-1.67) |  | 1.11 (0.70-1.76) |  |
|  | 4 | 31/215 | 1.27 (0.75-2.15) |  | 1.25 (0.74-2.12) |  | 1.12 (0.65-1.90) |  | 1.06 (0.62-1.80) |  |
|  | 5 | 5/ 40 | 1.29 (0.49-3.38) |  | 1.29 (0.49-3.40) |  | 1.12 (0.42-2.95) |  | 1.04 (0.40-2.76) |  |
| **African Men**  **(N=1,102)** | 0-1 | 17/151 | Reference | 0.379 | Reference | 0.431 | Reference | 0.430 | Reference | 0.664 |
|  | 2 | 64/384 | 1.63 (0.96-2.79) |  | 1.62 (0.95-2.76) |  | 1.62 (0.94-2.77) |  | 1.54 (0.90-2.63) |  |
|  | 3 | 68/415 | 1.56 (0.91-2.65) |  | 1.53 (0.90-2.60) |  | 1.51 (0.89-2.57) |  | 1.45 (0.85-2.47) |  |
|  | 4 | 21/134 | 1.42 (0.75-2.69) |  | 1.39 (0.73-2.64) |  | 1.41 (0.74-2.67) |  | 1.23 (0.64-2.35) |  |
|  | 5 | 3/ 18 | 1.81 (0.53-6.17) |  | 1.73 (0.51-5.92) |  | 1.72 (0.50-5.90) |  | 1.66 (0.48-5.72) |  |
| ^a^Model 1 includes age | | | | | | | | | | |
| ^b^Model 2 includes variables in Model 1 and household income and education | | | | | | | | | | |
| ^c^Model 3 includes variables in Model 2 and smoking status, alcohol consumption and physical activity | | | | | | | | | | |
| ^d^Model 4 includes variables in Model 3 and body mass index (BMI) | | | | | | | | | | |

**Supplemental Table 8.** Prospective associations between Circadian Imbalance Index (CII) and risk of incident Cardiovascular-Kidney-Metabolic for European ancestry UK biobank participants who reported the lifetime occupational history, stratified by duration of night shift work (n = 47,843).

| **Group** | **CII** | **Case/N** | **Model 1^a^** | | **Model 2^b^** | | **Model 3^c^** | | **Model 4^d^** | |
| --- | --- | --- | --- | --- | --- | --- | --- | --- | --- | --- |
|  |  |  | **HR (95%CI)** | ***P*_trend_** | **HR (95%CI)** | ***P*_trend_** | **HR (95%CI)** | ***P*_trend_** | **HR (95%CI)** | ***P*_trend_** |
| **Day workers**  **(N=36,111)** | 0-1 | 765/13 961 | Reference | <0.001 | Reference | <0.001 | Reference | <0.001 | Reference | <0.001 |
|  | 2 | 770/12 524 | 1.21 (1.09-1.34) |  | 1.20 (1.09-1.33) |  | 1.16 (1.05-1.29) |  | 1.08 (0.97-1.19) |  |
|  | 3 | 515/ 7 161 | 1.50 (1.34-1.68) |  | 1.48 (1.32-1.65) |  | 1.39 (1.24-1.56) |  | 1.26 (1.12-1.41) |  |
|  | 4 | 153/ 2 150 | 1.62 (1.36-1.93) |  | 1.59 (1.34-1.90) |  | 1.47 (1.24-1.75) |  | 1.23 (1.03-1.47) |  |
|  | 5 | 30/ 315 | 2.39 (1.66-3.45) |  | 2.35 (1.63-3.38) |  | 2.06 (1.43-2.98) |  | 1.68 (1.16-2.43) |  |
| <**10 years**  **(N=6,450)** | 0-1 | 185/2 411 | Reference | 0.009 | Reference | 0.019 | Reference | 0.089 | Reference | 0.480 |
|  | 2 | 191/2 197 | 1.24 (1.01-1.52) |  | 1.23 (1.01-1.51) |  | 1.20 (0.98-1.47) |  | 1.14 (0.93-1.40) |  |
|  | 3 | 104/1 341 | 1.17 (0.92-1.49) |  | 1.14 (0.90-1.45) |  | 1.08 (0.85-1.38) |  | 1.00 (0.78-1.27) |  |
|  | 4 | 40/ 442 | 1.57 (1.11-2.21) |  | 1.54 (1.09-2.17) |  | 1.41 (1.00-2.00) |  | 1.26 (0.89-1.79) |  |
|  | 5 | 6/ 59 | 1.69 (0.75-3.82) |  | 1.59 (0.71-3.60) |  | 1.38 (0.61-3.14) |  | 1.01 (0.44-2.31) |  |
| \| **10-20 years** \| \| --- \| \| **(N=2,290)** \| | 0-1 | 66/828 | Reference | 0.007 | Reference | 0.011 | Reference | 0.018 | Reference | 0.215 |
|  | 2 | 60/799 | 1.01 (0.71-1.43) |  | 1.00 (0.70-1.42) |  | 0.99 (0.70-1.41) |  | 0.91 (0.64-1.29) |  |
|  | 3 | 42/465 | 1.27 (0.86-1.88) |  | 1.25 (0.85-1.85) |  | 1.23 (0.83-1.81) |  | 1.00 (0.67-1.48) |  |
|  | 4 | 14/158 | 1.38 (0.77-2.47) |  | 1.34 (0.75-2.40) |  | 1.29 (0.72-2.33) |  | 1.12 (0.62-2.02) |  |
|  | 5 | 9/ 40 | 3.46 (1.72-6.94) |  | 3.30 (1.64-6.65) |  | 3.22 (1.58-6.57) |  | 2.28 (1.11-4.70) |  |
| **> 20 years**  **(N=2,992)** | 0-1 | 83/1 056 | Reference | <0.001 | Reference | <0.001 | Reference | <0.001 | Reference | <0.001 |
|  | 2 | 106/1 004 | 1.45 (1.08-1.93) |  | 1.45 (1.08-1.93) |  | 1.42 (1.06-1.89) |  | 1.28 (0.96-1.72) |  |
|  | 3 | 78/ 654 | 1.78 (1.31-2.43) |  | 1.73 (1.27-2.37) |  | 1.70 (1.24-2.32) |  | 1.55 (1.14-2.12) |  |
|  | 4 | 35/ 245 | 2.32 (1.56-3.45) |  | 2.26 (1.52-3.36) |  | 2.12 (1.42-3.16) |  | 1.94 (1.29-2.89) |  |
|  | 5 | 9/ 33 | 4.48 (2.25-8.92) |  | 4.25 (2.13-8.47) |  | 3.89 (1.94-7.82) |  | 3.12 (1.55-6.31) |  |
| ^a^Model 1 includes sex and age | | | | | | | | | | |
| ^b^Model 2 includes variables in Model 1 and household income and education | | | | | | | | | | |
| ^c^Model 3 includes variables in Model 2 and smoking status, alcohol consumption and physical activity | | | | | | | | | | |
| ^d^Model 4 includes variables in Model 3 and body mass index (BMI) | | | | | | | | | | |

**Supplemental Table 9.** Prospective associations between Circadian Health Index (CII) and risk of incident Cardiovascular-Kidney-Metabolic disease for European ancestry UK biobank participants who reported on the lifetime occupational history, stratified by the intensity of night shift work (n = 47,843).

| **Group** | **CII** | **Case/N** | **Model 1^a^** | | **Model 2^b^** | | **Model 3^c^** | | **Model 4^d^** | |
| --- | --- | --- | --- | --- | --- | --- | --- | --- | --- | --- |
|  |  |  | **HR (95%CI)** | ***P*_trend_** | **HR (95%CI)** | ***P*_trend_** | **HR (95%CI)** | ***P*_trend_** | **HR (95%CI)** | ***P*_trend_** |
| **Day workers**  **(N=36,111)** | 0-1 | 765/13 961 | Reference | <0.001 | Reference | <0.001 | Reference | <0.001 | Reference | <0.001 |
|  | 2 | 770/12 524 | 1.21 (1.09-1.34) |  | 1.20 (1.09-1.33) |  | 1.16 (1.05-1.29) |  | 1.08 (0.97-1.19) |  |
|  | 3 | 515/ 7 161 | 1.50 (1.34-1.68) |  | 1.48 (1.32-1.65) |  | 1.39 (1.24-1.56) |  | 1.26 (1.12-1.41) |  |
|  | 4 | 153/ 2 150 | 1.62 (1.36-1.93) |  | 1.59 (1.34-1.90) |  | 1.47 (1.24-1.75) |  | 1.23 (1.03-1.47) |  |
|  | 5 | 30/ 315 | 2.39 (1.66-3.45) |  | 2.35 (1.63-3.38) |  | 2.06 (1.43-2.98) |  | 1.68 (1.16-2.43) |  |
| **<3 night shifts/month**  **(N=1,575)** | 0-1 | 49/615 | Reference | 0.318 | Reference | 0.325 | Reference | 0.453 | Reference | 0.702 |
|  | 2 | 54/531 | 1.41 (0.96- 2.08) |  | 1.46 (0.99- 2.16) |  | 1.44 (0.98- 2.13) |  | 1.44 (0.97-2.13) |  |
|  | 3 | 27/310 | 1.21 (0.76- 1.94) |  | 1.19 (0.74- 1.91) |  | 1.13 (0.71- 1.82) |  | 1.09 (0.68-1.75) |  |
|  | 4 | 7/106 | 1.02 (0.46- 2.26) |  | 1.03 (0.47- 2.29) |  | 0.98 (0.44- 2.18) |  | 0.94 (0.42-2.10) |  |
|  | 5 | 2/ 13 | 3.08 (0.74-12.72) |  | 3.15 (0.76-13.05) |  | 3.09 (0.74-12.91) |  | 1.67 (0.38-7.26) |  |
| **3-8 night shifts/month**  **(N=4,992)** | 0-1 | 129/1 875 | Reference | 0.002 | Reference | 0.004 | Reference | 0.016 | Reference | 0.402 |
|  | 2 | 135/1 693 | 1.26 (0.99-1.61) |  | 1.27 (0.99-1.61) |  | 1.24 (0.97-1.58) |  | 1.11 (0.87-1.42) |  |
|  | 3 | 73/1 026 | 1.22 (0.91-1.62) |  | 1.19 (0.89-1.58) |  | 1.14 (0.85-1.52) |  | 0.94 (0.70-1.26) |  |
|  | 4 | 29/ 339 | 1.57 (1.05-2.36) |  | 1.54 (1.03-2.31) |  | 1.44 (0.96-2.17) |  | 1.15 (0.76-1.74) |  |
|  | 5 | 10/ 59 | 2.90 (1.52-5.52) |  | 2.66 (1.39-5.07) |  | 2.47 (1.29-4.73) |  | 1.90 (0.99-3.65) |  |
| **>8 night shifts/month**  **(N=5,165)** | 0-1 | 156/1 805 | Reference | <0.001 | Reference | <0.001 | Reference | <0.001 | Reference | <0.001 |
|  | 2 | 168/1 776 | 1.18 (0.95-1.47) |  | 1.17(0.94-1.46) |  | 1.15 (0.93-1.44) |  | 1.06 (0.85-1.32) |  |
|  | 3 | 124/1 124 | 1.47 (1.16-1.86) |  | 1.43(1.13-1.81) |  | 1.38 (1.09-1.75) |  | 1.28 (1.01-1.62) |  |
|  | 4 | 53/ 400 | 2.07 (1.51-2.83) |  | 1.99(1.45-2.73) |  | 1.89 (1.37-2.60) |  | 1.75 (1.27-2.41) |  |
|  | 5 | 12/ 60 | 3.04 (1.69-5.47) |  | 2.84(1.58-5.13) |  | 2.70 (1.49-4.89) |  | 2.08 (1.14-3.79) |  |
| ^a^Model 1 includes sex and age | | | | | | | | | | |
| ^b^Model 2 includes variables in Model 1 and household income and education | | | | | | | | | | |
| ^c^Model 3 includes variables in Model 2 and smoking status, alcohol consumption and physical activity | | | | | | | | | | |
| ^d^Model 4 includes variables in Model 3 and body mass index (BMI) | | | | | | | | | | |

**Supplemental Table 10.** Multivariable adjusted HRs with 95% CI and RERI, AP for additive interaction between Circadian Imbalance Index (CII) and lifetime duration years of night shift work for cardiovascular-kidney-metabolic disease risk among UKB participants European ancestry with occupational history, n = 47, 843

| **Characteristic** | **N** | **Case** | **HR (95% CI)**^1^ | ***P* value** | **RERI (95%CI)**^2^ | **AP (95%CI)**^3^ |
| --- | --- | --- | --- | --- | --- | --- |
| **Low CII (0-1)** |  |  |  |  |  |  |
| Day workers | 13,961 | 765 | Reference |  | Reference | Reference |
| Night shift work< 20 years | 3,239 | 251 | 1.36 (1.18, 1.57) | <0.001 | - | - |
| Night shift work>= 20 years | 1,056 | 83 | 1.20 (0.96, 1.51) | 0.11 | - | - |
| **Middle CII (2-3)** |  |  |  |  |  |  |
| Day workers | 19,685 | 1,285 | 1.30 (1.19, 1.42) | <0.001 | - | - |
| Night shift work< 20 years | 4,802 | 397 | 1.59 (1.40, 1.79) | <0.001 | -0.072 (-0.324 - 0.18) | -0.046 (-0.206 - 0.115) |
| Night shift work>= 20 years | 1,658 | 184 | 1.89 (1.60, 2.22) | <0.001 | **0.387 (0.001 - 0.772)** | **0.205 (0.018 - 0.391)** |
| **High CII (4-5)** |  |  |  |  |  |  |
| Day workers | 2,465 | 183 | 1.68 (1.43, 1.98) | <0.001 | - | - |
| Night shift work< 20 years | 699 | 69 | 2.17 (1.69, 2.78) | <0.001 | 0.128 (-0.467 - 0.722) | 0.059 (-0.204 - 0.322) |
| Night shift work>= 20 years | 278 | 44 | 3.04 (2.24, 4.13) | <0.001 | **1.162 (0.194 - 2.131)** | **0.382 (0.164 - 0.600)** |

^1^HR = Hazard Ratio, CI = Confidence Interval

^2^RERI = relative excess risk due to the interaction

^3^AP = attributable proportion due to the interaction

^*^Model adjusted for sex, age, household income, education and recruitment season

^*^To estimate the RERI and AP, the low Circadian Imbalance Index (0-1) and the day worker group were the reference categories.

**Supplemental Table 11.** Multivariable adjusted hazard ratios with 95% CI and RERI, AP for additive interaction between Circadian Imbalance Index (CII) and intensity of night shift work for cardiovascular-kidney-metabolic disease risk among UKB participants European ancestry with occupational history, n = 47, 843

| **Characteristic** | **N** | **Case** | **HR (95% CI)**^1^ | ***P* value** | **RERI (95%CI)**^2^ | **AP (95%CI)**^3^ |
| --- | --- | --- | --- | --- | --- | --- |
| **Low CII (0-1)** |  |  |  |  |  |  |
| Day workers | 13,961 | 765 | Reference |  | Reference | Reference |
| < 8 nights/month | 2,490 | 178 | 1.27 (1.08, 1.50) | 0.004 | - | - |
| >= 8 nights/month | 1,805 | 156 | 1.37 (1.15, 1.63) | <0.001 | - | - |
| **Middle CII (2-3)** |  |  |  |  |  |  |
| Day workers | 19,685 | 1,285 | 1.30 (1.19, 1.42) | <0.001 | - | - |
| < 8 nights/month | 3,560 | 289 | 1.61 (1.41, 1.85) | <0.001 | 0.039 (-0.242 - 0.320) | 0.024 (-0.149 - 0.197) |
| >= 8 nights/month | 2,900 | 292 | 1.74 (1.52, 1.99) | <0.001 | 0.065 (-0.244 - 0.374) | 0.037 (-0.138 - 0.213) |
| **High CII (4-5)** |  |  |  |  |  |  |
| Day workers | 2,465 | 183 | 1.68 (1.43, 1.98) | <0.001 | - | - |
| < 8 nights/month | 517 | 48 | 2.03 (1.51, 2.72) | <0.001 | 0.077 (-0.578 - 0.731) | 0.038 (-0.276 - 0.351) |
| >= 8 nights/month | 460 | 65 | 2.87 (2.23, 3.71) | <0.001 | **0.822 (0.049 - 1.595)** | **0.286 (0.077 - 0.495)** |

^1^HR = Hazard Ratio, CI = Confidence Interval

^2^RERI = relative excess risk due to the interaction

^3^AP = attributable proportion due to the interaction

^*^Model adjusted for sex, age, household income, education and recruitment season

^*^To estimate the RERI and AP, the low clock risk score category (0-1) and the day worker group were the reference categories.

**Supplemental Table 12.** Prospective associations between Circadian Imbalance Index (CII) and risk of incident type 2 diabetes (T2D) for participants from the UK Biobank, stratified by genetic ethnicity

| **Ethnicity** | **CII** | **Case / N** | **Model 1^a^** | | **Model 2^b^** | | **Model 3^c^** | | **Model 4^d^** | |
| --- | --- | --- | --- | --- | --- | --- | --- | --- | --- | --- |
|  |  |  | **HR (95%CI)** | ***P*_trend_** | **HR (95%CI)** | ***P*_trend_** | **HR (95%CI)** | ***P*_trend_** | **HR(95%CI)** | ***P*_trend_** |
| **European**  **(N= 166,194)** | 0-1 | 1,709 / 59,921 | Reference | <0.001 | Reference | <0.001 | Reference | <0.001 | Reference | <0.001 |
|  | 2 | 2,117 / 56,698 | 1.39 (1.31-1.49) |  | 1.37 (1.29-1.46) |  | 1.31 (1.23-1.40) |  | 1.16 (1.09-1.24) |  |
|  | 3 | 1,625 / 35,600 | 1.80 (1.68-1.93) |  | 1.73 (1.61-1.85) |  | 1.60 (1.49-1.71) |  | 1.33 (1.24-1.43) |  |
|  | 4 | 647 / 12,058 | 2.25 (2.05-2.46) |  | 2.11 (1.93-2.31) |  | 1.90 (1.74-2.09) |  | 1.49 (1.36-1.64) |  |
|  | 5 | 126 / 1,917 | 2.97 (2.48-3.57) |  | 2.74 (2.29-3.29) |  | 2.37 (1.98-2.85) |  | 1.65 (1.37-1.98) |  |
| **Asian**  **(N= 3,481)** | 0-1 | 47 / 562 | Reference | 0.002 | Reference | 0.003 | Reference | 0.011 | Reference | 0.045 |
|  | 2 | 160 / 1,350 | 1.51 (1.09-2.09) |  | 1.50 (1.09-2.08) |  | 1.49 (1.07-2.06) |  | 1.45 (1.05-2.01) |  |
|  | 3 | 142 / 1,103 | 1.65 (1.19-2.30) |  | 1.64 (1.18-2.29) |  | 1.58 (1.13-2.19) |  | 1.47 (1.05-2.05) |  |
|  | 4 | 50 / 398 | 1.74 (1.17-2.59) |  | 1.67 (1.12-2.49) |  | 1.60 (1.07-2.40) |  | 1.48 (0.99-2.21) |  |
|  | 5 | 9 / 68 | 2.40 (1.17-4.93) |  | 2.26 (1.10-4.64) |  | 2.04 (0.99-4.20) |  | 1.98 (0.96-4.09) |  |
| **African**  **(N= 2,583)** | 0-1 | 30 / 333 | Reference | 0.493 | Reference | 0.510 | Reference | 0.599 | Reference | 0.902 |
|  | 2 | 106 / 897 | 1.44 (0.96-2.16) |  | 1.42 (0.95-2.13) |  | 1.36 (0.90-2.04) |  | 1.31 (0.88-1.98) |  |
|  | 3 | 98 / 946 | 1.25 (0.83-1.89) |  | 1.24 (0.82-1.86) |  | 1.18 (0.78-1.78) |  | 1.18 (0.78-1.78) |  |
|  | 4 | 39 / 349 | 1.35 (0.84-2.17) |  | 1.35 (0.84-2.17) |  | 1.29 (0.80-2.08) |  | 1.15 (0.71-1.86) |  |
|  | 5 | 6 / 58 | 1.51 (0.63-3.64) |  | 1.48 (0.62-3.58) |  | 1.46 (0.60-3.52) |  | 1.29 (0.53-3.12) |  |
| ^a^Model 1 includes sex and age | | | | | | | | | | |
| ^b^Model 2 includes variables in Model 1 and household income and education | | | | | | | | | | |
| ^c^Model 3 includes variables in Model 2 and smoking status, alcohol consumption and physical activity | | | | | | | | | | |
| ^d^Model 4 includes variables in Model 3 and body mass index (BMI) | | | | | | | | | | |

**Supplemental Table 13.** Prospective associations between Circadian Imbalance Index (CII) and risk of incident chronic cardiovascular diseases (CVD) for participants from the UK Biobank, stratified by genetic ethnicity

| **Ethnicity** | **CII** | **Case / N** | **Model 1^a^** | | **Model 2^b^** | | **Model 3^c^** | | **Model 4^d^** | |
| --- | --- | --- | --- | --- | --- | --- | --- | --- | --- | --- |
|  |  |  | **HR (95%CI)** | ***P*_trend_** | **HR (95%CI)** | ***P*_trend_** | **HR (95%CI)** | ***P*_trend_** | **HR(95%CI)** | ***P*_trend_** |
| **European**  **(N= 166,194)** | 0-1 | 2,412 / 59,921 | Reference | <0.001 | Reference | <0.001 | Reference | <0.001 | Reference | <0.001 |
|  | 2 | 2,594 / 56,698 | 1.23 (1.17-1.30) |  | 1.22 (1.15-1.29) |  | 1.19 (1.12-1.26) |  | 1.15 (1.09-1.22) |  |
|  | 3 | 1,666 / 35,600 | 1.34 (1.26-1.43) |  | 1.31 (1.23-1.39) |  | 1.25 (1.17-1.33) |  | 1.19 (1.11-1.26) |  |
|  | 4 | 569 / 12,058 | 1.46 (1.33-1.60) |  | 1.40 (1.28-1.54) |  | 1.31 (1.19-1.43) |  | 1.22 (1.11-1.34) |  |
|  | 5 | 99 / 1,917 | 1.76 (1.44-2.16) |  | 1.67 (1.37-2.04) |  | 1.53 (1.25-1.87) |  | 1.37 (1.12-1.68) |  |
| **Asian**  **(N= 3,481)** | 0-1 | 27 / 562 | Reference | 0.365 | Reference | 0.418 | Reference | 0.500 | Reference | 0.614 |
|  | 2 | 66 / 1,350 | 1.09 (0.70-1.71) |  | 1.08 (0.69-1.70) |  | 1.06 (0.68-1.67) |  | 1.05 (0.67-1.64) |  |
|  | 3 | 52 / 1,103 | 1.05 (0.66-1.67) |  | 1.04 (0.65-1.65) |  | 1.01 (0.63-1.61) |  | 0.98 (0.61-1.56) |  |
|  | 4 | 23 / 398 | 1.45 (0.83-2.54) |  | 1.42 (0.81-2.48) |  | 1.36 (0.78-2.39) |  | 1.30 (0.74-2.29) |  |
|  | 5 | 2 / 68 | 1.03 (0.24-4.36) |  | 0.99 (0.23-4.19) |  | 0.99 (0.23-4.21) |  | 0.96 (0.23-4.09) |  |
| **African**  **(N= 2,583)** | 0-1 | 14 / 333 | Reference | 0.368 | Reference | 0.418 | Reference | 0.443 | Reference | 0.540 |
|  | 2 | 32 / 897 | 0.94 (0.50-1.77) |  | 0.93 (0.50-1.75) |  | 0.91 (0.48-1.71) |  | 0.88 (0.47-1.66) |  |
|  | 3 | 37 / 946 | 1.04 (0.56-1.92) |  | 1.01 (0.55-1.87) |  | 0.99 (0.53-1.83) |  | 0.98 (0.53-1.82) |  |
|  | 4 | 15 / 349 | 1.19 (0.57-2.46) |  | 1.17 (0.56-2.42) |  | 1.17 (0.56-2.43) |  | 1.08 (0.52-2.24) |  |
|  | 5 | 3 / 58 | 1.85 (0.53-6.48) |  | 1.76 (0.50-6.17) |  | 1.56 (0.44-5.52) |  | 1.47 (0.41-5.21) |  |
| ^a^Model 1 includes sex and age | | | | | | | | | | |
| ^b^Model 2 includes variables in Model 1 and household income and education | | | | | | | | | | |
| ^c^Model 3 includes variables in Model 2 and smoking status, alcohol consumption and physical activity | | | | | | | | | | |
| ^d^Model 4 includes variables in Model 3 and body mass index (BMI) | | | | | | | | | | |

**Supplemental Table 14.** Prospective associations between Circadian Imbalance Index (CII) and risk of incident chronic kidney diseases (CKD) for participants from the UK Biobank, stratified by genetic ethnicity

| **Ethnicity** | **CII** | **Case / N** | **Model 1^a^** | | **Model 2^b^** | | **Model 3^c^** | | **Model 4^d^** | |
| --- | --- | --- | --- | --- | --- | --- | --- | --- | --- | --- |
|  |  |  | **HR (95%CI)** | ***P*_trend_** | **HR (95%CI)** | ***P*_trend_** | **HR (95%CI)** | ***P*_trend_** | **HR(95%CI)** | ***P*_trend_** |
| **European**  **(N= 166,194)** | 0-1 | 959 / 59,921 | Reference | <0.001 | Reference | <0.001 | Reference | <0.001 | Reference | <0.001 |
|  | 2 | 990 / 56,698 | 1.18 (1.08-1.29) |  | 1.16 (1.06-1.27) |  | 1.13 (1.03-1.23) |  | 1.07 (0.98-1.17) |  |
|  | 3 | 615 / 35,600 | 1.24 (1.12-1.37) |  | 1.20 (1.08-1.32) |  | 1.13 (1.02-1.26) |  | 1.05 (0.94-1.16) |  |
|  | 4 | 265 / 12,058 | 1.70 (1.49-1.95) |  | 1.62 (1.41-1.86) |  | 1.51 (1.31-1.73) |  | 1.36 (1.19-1.56) |  |
|  | 5 | 42 / 1,917 | 1.89 (1.39-2.57) |  | 1.77 (1.30-2.42) |  | 1.61 (1.18-2.19) |  | 1.35 (0.99-1.85) |  |
| **Asian**  **(N= 3,481)** | 0-1 | 11 / 562 | Reference | 0.956 | Reference | 0.957 | Reference | 0.990 | Reference | 0.905 |
|  | 2 | 23 / 1,350 | 0.94 (0.46-1.93) |  | 0.93 (0.45-1.90) |  | 0.90 (0.44-1.86) |  | 0.88 (0.43-1.82) |  |
|  | 3 | 15 / 1,103 | 0.76 (0.35-1.65) |  | 0.75 (0.34-1.63) |  | 0.74 (0.34-1.61) |  | 0.71 (0.32-1.55) |  |
|  | 4 | 9 / 398 | 1.47 (0.61-3.57) |  | 1.38 (0.57-3.34) |  | 1.41 (0.58-3.44) |  | 1.33 (0.54-3.24) |  |
|  | 5 | 0 / 68 | -- |  | -- |  | -- |  | -- |  |
| **African**  **(N= 2,583)** | 0-1 | 9 / 333 | Reference | 0.231 | Reference | 0.248 | Reference | 0.346 | Reference | 0.368 |
|  | 2 | 27 / 897 | 1.24 (0.58-2.64) |  | 1.26 (0.59-2.68) |  | 1.19 (0.56-2.56) |  | 1.17 (0.55-2.52) |  |
|  | 3 | 40 / 946 | 1.81 (0.88-3.74) |  | 1.81 (0.88-3.74) |  | 1.71 (0.83-3.55) |  | 1.72 (0.83-3.56) |  |
|  | 4 | 12 / 349 | 1.43 (0.60-3.40) |  | 1.43 (0.60-3.40) |  | 1.33 (0.56-3.16) |  | 1.28 (0.54-3.06) |  |
|  | 5 | 1 / 58 | 0.96 (0.12-7.59) |  | 0.92 (0.12-7.31) |  | 0.79 (0.10-6.30) |  | 0.76 (0.09-6.10) |  |
| ^a^Model 1 includes sex and age | | | | | | | | | | |
| ^b^Model 2 includes variables in Model 1 and household income and education | | | | | | | | | | |
| ^c^Model 3 includes variables in Model 2 and smoking status, alcohol consumption and physical activity | | | | | | | | | | |
| ^d^Model 4 includes variables in Model 3 and body mass index (BMI) | | | | | | | | | | |

**Supplemental Table 15.** Prospective associations between individual circadian related traits being components of Circadian Imbalance Index (CII) and risk of incident Cardiovascular-Kidney-Metabolic disease for European ancestry participants from the UK Biobank, n = 166,194

|  | Model 1^a^ | | Model 2^b^ | | Model 3^c^ | | Model 4^d^ | |
| --- | --- | --- | --- | --- | --- | --- | --- | --- |
| Circadian traits | HR(95% CI) | *P*_value | HR(95% CI) | *P*_value | HR(95% CI) | *P*_value | HR(95% CI) | *P*_value |
| Evening type | 1.05 (1.01-1.09) | 0.006 | 1.05 (1.01-1.09) | 0.007 | 1.02 (0.99-1.06) | 0.184 | 1.02 (0.99-1.06) | 0.217 |
| Sleep long/short | 1.25 (1.21-1.29) | <0.001 | 1.21 (1.17-1.26) | <0.001 | 1.19 (1.15-1.24) | <0.001 | 1.12 (1.08-1.16) | <0.001 |
| Neuroticism >=7 | 1.17 (1.13-1.22) | <0.001 | 1.13 (1.09-1.18) | <0.001 | 1.12 (1.08-1.17) | <0.001 | 1.14 (1.10-1.19) | <0.001 |
| Atypical caffeinated coffee | 1.17 (1.13-1.21) | <0.001 | 1.13 (1.10-1.17) | <0.001 | 1.10 (1.06-1.13) | <0.001 | 1.08 (1.04-1.11) | <0.001 |
| Low vitamin D | 1.30 (1.26-1.34) | <0.001 | 1.32 (1.28-1.37) | <0.001 | 1.28 (1.24-1.33) | <0.001 | 1.11 (1.07-1.15) | <0.001 |
| ^a^Model 1 includes sex and age | | | | | | | | |
| ^b^Model 2 includes variables in Model 1 and household income and education | | | | | | | | |
| ^c^Model 3 includes variables in Model 2 and smoking status, alcohol consumption and physical activity | | | | | | | | |
| ^d^Model 4 includes variables in Model 3 and body mass index (BMI) | | | | | | | | |

*For each model, the individual trait was mutually adjusted for the rest of other circadian related traits.

**Supplemental Table 16.** Prospective associations between Circadian Imbalance Index (CII) and risk of incident Cardiovascular-Kidney-Metabolic disease: a cause-specific transition analysis stratified by genetic ethnicity in the UK Biobank

|  |  | **Type 2 Diabetes (T2D)^a^** | | | **Cardiovascular Disease (CVD)^a^** | | | **Chronic Kidney Disease (CKD)^a^** | | |
| --- | --- | --- | --- | --- | --- | --- | --- | --- | --- | --- |
| **Ethnicity** | **CII** | **Events/N** | **csHR**  **(95%CI)** | ***P*_trend_** | **Events/N** | **csHR (95%CI)** | ***P*_trend_** | **Events/N** | **csHR (95%CI)** | ***P*_trend_** |
| **European**  **(N=166, 194)** | 0-1 | 1 624 / 59 921 | Ref. | <0.001 | 2 173 / 59 921 | Ref. | <0.001 | 677 / 59 921 | Ref. | <0.001 |
|  | 2 | 2 001 / 56 698 | 1.37 (1.28-1.46) |  | 2 270 / 56 698 | 1.19 (1.12-1.27) |  | 708 / 56 698 | 1.18 (1.06-1.31) |  |
|  | 3 | 1 551 / 35 600 | 1.74 (1.62-1.87) |  | 1 443 / 35 600 | 1.27 (1.19-1.36) |  | 400 / 35 600 | 1.10 (0.97-1.25) |  |
|  | 4 | 617 / 12 058 | 2.13 (1.94-2.34) |  | 478 / 12 058 | 1.33 (1.21-1.47) |  | 174 / 12 058 | 1.49 (1.26-1.76) |  |
|  | 5 | 115 / 1 917 | 2.63 (2.18-3.18) |  | 80 / 1 917 | 1.53 (1.22-1.91) |  | 26 / 1 917 | 1.53 (1.03-2.27) |  |
| **Asian**  **(N=3, 481)** | 0-1 | 45 / 562 | Ref. | 0.003 | 19 / 562 | Ref. | 0.307 | 3 / 562 | Ref. | 0.812 |
|  | 2 | 150 / 1 350 | 1.48 (1.06-2.07) |  | 48 / 1 350 | 1.16 (0.68-1.97) |  | 13 / 1 350 | 1.98 (0.56- 6.94) |  |
|  | 3 | 141 / 1 103 | 1.70 (1.22-2.38) |  | 36 / 1 103 | 1.04 (0.60-1.81) |  | 6 / 1 103 | 1.12 (0.28- 4.50) |  |
|  | 4 | 46 / 398 | 1.63 (1.08-2.46) |  | 18 / 398 | 1.60 (0.84-3.07) |  | 4 / 398 | 2.34 (0.52-10.55) |  |
|  | 5 | 9 / 68 | 2.38 (1.16-4.90) |  | 2 / 68 | 1.44 (0.33-6.26) |  | 0 / 68 | -- |  |
| **African**  **(N= 2,583)** | 0-1 | 29 / 333 | Ref. | 0.758 | 8 / 333 | Ref. | 0.164 | 5 / 333 | Ref. | 0.806 |
|  | 2 | 101 / 897 | 1.41 (0.93-2.13) |  | 22 / 897 | 1.15 (0.51-2.59) |  | 18 / 897 | 1.60 (0.59-4.32) |  |
|  | 3 | 94 / 946 | 1.23 (0.81-1.87) |  | 26 / 946 | 1.26 (0.57-2.78) |  | 22 / 946 | 1.83 (0.69-4.84) |  |
|  | 4 | 33 / 349 | 1.19 (0.72-1.96) |  | 13 / 349 | 1.74 (0.72-4.21) |  | 6 / 349 | 1.35 (0.41-4.43) |  |
|  | 5 | 6 / 58 | 1.55 (0.64-3.74) |  | 2 / 58 | 1.91 (0.40-9.04) |  | 0 / 58 | -- |  |

^a^Model includes variables age, sex, household income and education

**Supplemental Table 17.** Prospective associations between Circadian Imbalance Index (CII) and risk of incident Cardiovascular-Kidney-Metabolic disease among 2,583 African ancestry participants from the UK Biobank.

| **Ethnicity** | **CII** | **Case/N** | **Absolute Event Rates** | **Model 1^a^** | | **Model 2^b^** | | **Model 3^c^** | | **Model 4^d^** | |
| --- | --- | --- | --- | --- | --- | --- | --- | --- | --- | --- | --- |
|  |  |  | **(events/1000PY)** | **HR (95%CI)** | ***P*_trend_** | **HR (95%CI)** | ***P*_trend_** | **HR (95%CI)** | ***P*_trend_** | **HR (95%CI)** | ***P*_trend_** |
| **African**  **(N= 2,583)** | 0-1 | 42/333 | 10.55 | Reference | 0.326 | Reference | 0.353 | Reference | 0.442 | Reference | 0.662 |
|  | 2 | 141/897 | 13.35 | 1.39(0.99-1.96) |  | 1.38(0.98-1.95) |  | 1.33(0.94-1.87) |  | 1.29(0.91-1.82) |  |
|  | 3 | 142/946 | 12.66 | 1.32(0.94-1.86) |  | 1.30(0.92-1.84) |  | 1.25(0.89-1.77) |  | 1.26(0.89-1.78) |  |
|  | 4 | 52/349 | 12.42 | 1.32(0.88-1.98) |  | 1.31(0.87-1.97) |  | 1.27(0.84-1.90) |  | 1.16(0.77-1.74) |  |
|  | 5 | 8/ 58 | 12.27 | 1.46(0.68-3.11) |  | 1.43(0.67-3.06) |  | 1.36(0.63-2.91) |  | 1.24(0.58-2.65) |  |
| ^a^Model 1 includes sex and age | | | | | | | | | | | |
| ^b^Model 2 includes variables in Model 1 and household income and education | | | | | | | | | | | |
| ^c^Model 3 includes variables in Model 2 and smoking status, alcohol consumption and physical activity | | | | | | | | | | | |
| ^d^Model 4 includes variables in Model 3 and body mass index (BMI) | | | | | | | | | | | |

**Reference:**

1. Karczewski, K.J., et al., *Pan-UK Biobank GWAS improves discovery, analysis of genetic architecture, and resolution into ancestry-enriched effects.* medRxiv, 2024: p. 2024.03.13.24303864.

2. *Pan UKBB website*. Available from: <https://pan.ukbb.broadinstitute.org/>.

3. Li, R. and L. Chambless, *Test for additive interaction in proportional hazards models.* Ann Epidemiol, 2007. **17**(3): p. 227-36.
